# Modular Synthetic Approach to Carboranyl‒Biomolecules Conjugates

**DOI:** 10.3390/molecules26072057

**Published:** 2021-04-03

**Authors:** Martin Kellert, Jan-Simon Jeshua Friedrichs, Nadine Anke Ullrich, Alexander Feinhals, Jonas Tepper, Peter Lönnecke, Evamarie Hey-Hawkins

**Affiliations:** Institute of Inorganic Chemistry, Faculty of Chemistry and Mineralogy, Leipzig University, Johannisallee 29, 04103 Leipzig, Germany; martin.kellert@uni-leipzig.de (M.K.); jf45guvu@studserv.uni-leipzig.de (J.-S.J.F.); nadine-ullrich6@web.de (N.A.U.); alexander.feinhals@gmx.de (A.F.); jonas.tepper@web.de (J.T.); loenneck@rz.uni-leipzig.de (P.L.)

**Keywords:** boron neutron cancer therapy (BNCT), modular approach, carboxylic acids and amines

## Abstract

The development of novel, tumor-selective and boron-rich compounds as potential agents for use in boron neutron capture therapy (BNCT) represents a very important field in cancer treatment by radiation therapy. Here, we report the design and synthesis of two promising compounds that combine *meta*-carborane, a water-soluble monosaccharide and a linking unit, namely glycine or ethylenediamine, for facile coupling with various tumor-selective biomolecules bearing a free amino or carboxylic acid group. In this work, coupling experiments with two selected biomolecules, a coumarin derivative and folic acid, were included. The task of every component in this approach was carefully chosen: the carborane moiety supplies ten boron atoms, which is a tenfold increase in boron content compared to the l-boronophenylalanine (l-BPA) presently used in BNCT; the sugar moiety compensates for the hydrophobic character of the carborane; the linking unit, depending on the chosen biomolecule, acts as the connection between the tumor-selective component and the boron-rich moiety; and the respective tumor-selective biomolecule provides the necessary selectivity. This approach makes it possible to develop a modular and feasible strategy for the synthesis of readily obtainable boron-rich agents with optimized properties for potential applications in BNCT.

## 1. Introduction

Since boron neutron capture therapy (BNCT) was ascertained to be a very promising binary cancer treatment [1,2,3,4], research has focused on the development of potent and selective boron-containing drugs [5,6]. The main advantage of this therapy is the generation of highly cytotoxic particles comprising a high linear energy transfer (LET) (α particle and Li particle). Their free mean path lengths of about 5 to 9 µm [7,8] roughly represent the diameter of a human cell [5]; therefore, these particles can only harm the surrounding tissue within this radius. However, only the lighter isotope of boron, ^10^B (20% natural abundance) [9], produces high LET particles after irradiation with thermal neutrons [10]. Therefore, BNCT agents have to be enriched with ^10^B [11,12]. Activation of the BNCT agents is caused by irradiation with thermal neutrons [13,14] for which ^10^B exhibits a large capture cross section (3835 barn, 1 barn = 1 × 10^−24^ cm^2^) [9]. This renders BNCT a promising strategy to treat malignant tissue with tumor-selective boron-containing drugs [6,15,16,17,18,19,20], as the thermal neutron beam can be focused on the affected area [21,22,23,24], thus generating therapeutic particles only upon neutron irradiation. In this manner, normal tissue can be spared and severe side effects, as known from pure radiotherapy or systemic effective chemotherapy, can be reduced.

The first boron-containing compounds used in clinical trials were l-boronophenylalanine (l-BPA) and sodium borocaptate (BSH) [5,6,10], but both compounds exhibit several drawbacks. For example, BSH and BPA do not exhibit optimized selectivity towards cancer cells (especially BSH), show a limited solubility in water (especially BPA [25]) and, in the case of BSH, are not able to penetrate cells due to their anionic character. Therefore, their application follows a tailored strategy where BSH is mostly applied for glioma treatment, as the dianionic compound is able to cross the damaged blood–brain barrier adjoining the malignant tissue in the human brain, and BPA is used as its fructose complex to overcome the low water solubility [5,6,10,25]. Since May 2020, the company Stella Pharma [26] has been allowed to market Steboronine^®^ [27] (generic name: Borofalan), which contains ^10^B-enriched (99%) l-BPA as its d-sorbitol complex. This BNCT agent, in comparison to the respective fructose complex, exhibits the advantage of being storable for about three years and does not have to be freshly prepared for each use with retention of its GMP grade.

Therefore, the development of novel boron-containing tumor-selective agents for application in BNCT is important to overcome these limitations [19,20]. For all compounds, the basic requirements that must be fulfilled are: sufficient water solubility, low inherent toxicity, high boron content and high tumor selectivity. Water solubility can be increased by using charged compounds [28] or introducing hydrophilic moieties [29,30]. Tumor-selectivity can be achieved by using essential biological nutrients, substrates like boronated saccharides or amino acids [19,20,31], or even tumor-selective complex compounds, like boron-containing antibodies [20,29,30,32,33,34,35,36]. A variety of different boron-containing bioconjugates are known, including nucleosides [16], carbohydrates [37,38], amino acids [39,40,41] and peptides [29,30,42,43]. One main prerequisite of BNCT especially plays an essential role in this treatment, namely the selective accumulation of sufficient amounts of ^10^B-containing compounds in cancerous tissues, so that the therapeutically active particles destroy only the malignant cells without destroying healthy tissue. An effective treatment requires boron concentrations of 10–30 µg ^10^B/g tumor, or 10^9 10^B-atoms/cancer cell [7,10]. One approach focuses on compounds with a very large boron content [29,30,42,44,45], another on the use of very selective BNCT agents over a longer period, taking advantage of specific shuttle systems that facilitate accumulation of the compound in the cells by internalization processes [17,29,30,33,46]. We pursued a combination of both strategies by combining tumor-selective small peptides, such as highly selective G protein-coupled receptor agonists, as biomolecules with *meta*-carborane derivatives to increase the boron load [29,30,43,47,48]. However, very high carborane loading (more than two carboranes attached to a peptide including 36 amino acids) results in loss of solubility or aggregation in aqueous media and, therefore, decreased potency and higher EC_50_ values [29,30]. Carbohydrate moieties, such as galactosyl groups, can be employed to compensate the hydrophobic character of carborane clusters (up to eight modified carboranes attached to the same peptide comprising 36 amino acids).

**Scheme 1:**
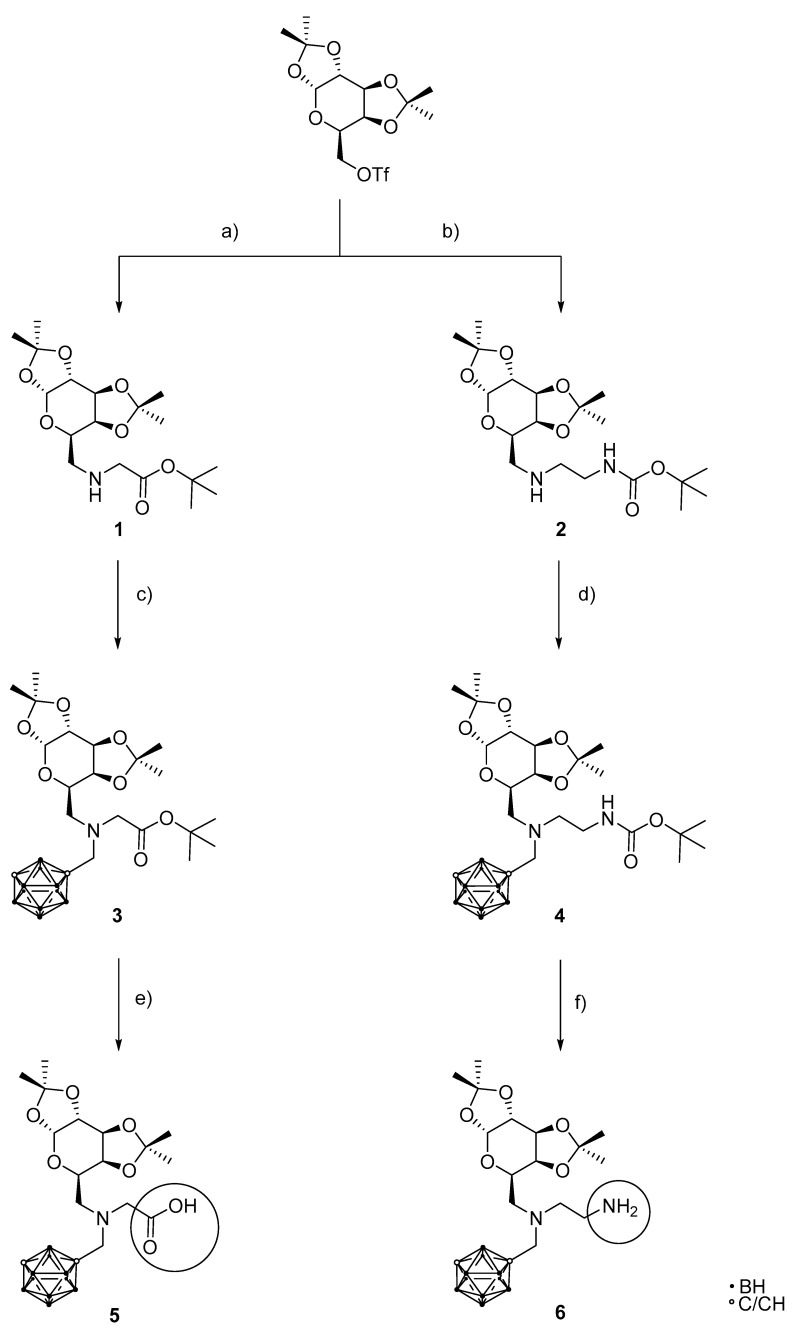
Synthesis of galactopyranosyl-modified *meta*-carborane-containing carboxylic acid (**5**) and primary amine (**6**) as the target compounds for modular conjugation with tumor-selective biomolecules. (**a**) 1.20 eq. *tert*-butyl glycinate hydrochloride, 2.96 eq. DIPEA, MeCN, 72 h, 45 °C, 68%; (**b**) 1.11 eq. *N*-*tert*-butoxycarbonyl-ethylenediamine, 1.12 eq. DIPEA, MeCN, 15 min, 0 °C, then 48 h, 40 °C, 77%; (**c**) 1.20 eq. 1-(trifluoromethanesulfonylmethyl)-1,7-dicarba-*closo*-dodeca-borane(12), 1.20 eq. K_2_CO_3_, toluene, 43 h, 95 °C, 54%; (**d**) 1.20 eq. 1-(trifluoromethanesulfonylmethyl)-1,7-dicarba-*closo*-dodecaborane(12), 1.20 eq. K_2_CO_3_, toluene, 48 h, 98 °C, 51%; (**e**) 55.0 eq. TFA, 3 h, rt, 65%; (**f**) 48.2 eq. TFA, 3 h, rt, quant.

Here, we report the development of small molecules representing potential boron-rich coupling partners for tumor-selective molecules based on a modular strategy [47,48,49] combining readily available starting materials, like *meta*-carborane, α-d-galactopyranose and glycine or ethylenediamine derivatives (compounds **5** and **6** in Scheme 1). Compounds bearing a primary amine or carboxylic acid group represent potentially universal coupling partners for biomolecules [48]; representative coupling experiments are also included here to demonstrate the generalizability of this approach.

In this regard, the synthesis of bifunctional anticancer drugs is of special interest. Several examples are known where drugs are used as theranostic compounds [50,51] or exhibit dual effects [52,53]. For example, derivatives of 7-amino-4-methylcoumarin are known for their anticancer activity [54]. Thus, combination with a carborane derivative can lead to drugs that possess anti-cancer properties and the ability to capture thermal neutrons for applications in BNCT. Furthermore, folic acid and its derivatives, which are already used as tumor-selective synthons for applications in BNCT [15,32,55], can act as diagnostic probes for some solid cancer types when combined with imaging agents [56]. Thus, conjugates of folic acid with carborane derivatives and an imaging agent using both carboxylic groups of folic acid could be used to generate highly selective BNCT agents with excellent imaging properties.

## 2. Results and Discussion

The starting materials 1-(trifluoromethanesulfonylmethyl)-1,7-dicarba-*closo*-dodecaborane(12) and 1,2:3,4-di-*O*-isopropylidene-6-deoxy-α-d-galactopyranosyl-6-triflate were prepared according to protocols from the literature. The synthetic procedures of Goto and co-workers for the synthesis of 1-(hydroxymethyl)-1,7-dicarba-*closo*-dodecaborane(12) [57] and those of Kalinin and co-workers for the preparation of 1-(trifluoromethanesulfonylmethyl)-1,7-dicarba-*closo*-dodecaborane(12) [58] were employed. 1,2:3,4-Di-*O*-isopropylidene-α-d-galactopyranose is commercially available or can be prepared in quantitative yield according to a procedure described by Saltan and co-workers [59]. 1,2:3,4-Di-*O*-isopropylidene-α-d-galactopyranose was converted to the triflate 1,2:3,4-di-*O*-isopropylidene-6-deoxy-α-d-galactopyranosyl-6-triflate following a procedure described by Brackhagen and co-workers [60].

The next step was the introduction of the respective linking units (glycine or ethylenediamine) starting from the commercially available protected derivatives, namely *tert*-butyl glycinate hydrochloride and *N*-*tert*-butoxycarbonyl-ethylenediamine, to prevent undesired side reactions during the synthetic steps. The reaction with the galactopyranosyl moiety was conducted under basic conditions using *N*,*N*-diisopropylethylamine (DIPEA) as a base in acetonitrile (MeCN) at elevated temperature (Scheme 1a,b). Both reactions gave the desired products, *tert*-butyl-*N*-(1,2:3,4-di-*O*-isopropyliden-6-deoxy-α-d-galactopyranos-6-yl)glycinate (**1**) and *tert*-butyl-{2-[(1,2:3,4-di-*O*-isopropylidene-6-deoxy-α-d-galactopyranos-6-yl)amino]ethyl}carbamate (**2**) in good to excellent yields.

Beside the desired product **2**, the disubstituted compound *tert*-butyl-(2-{[bis(1,2:3,4-di-*O*-isopropylidene-6-deoxy-α-d-galactopyranos-6-yl)]amino}ethyl)-carbamate (**2′**) was isolated with an 8% yield (see the Supplementary Materials). This product was formed due to the increased nucleophilicity of the secondary amine in **2** in comparison to a primary amine in the starting material; the amount corresponded to the small excess of the ethylenediamine derivative employed here.

The carboranyl moiety was introduced by reacting **1** and **2** with 1-(trifluoromethanesulfonylmethyl)-1,7-dicarba-*closo*-dodecaborane(12) under basic conditions (potassium carbonate) in toluene (Scheme 1c,d); for optimized reaction conditions see the Supplementary Materials. The products, *tert*-butyl-*N*-[(1,7-dicarba-*closo*-dodecaboran-1-yl)methyl]-*N*-(1,2:3,4-di-*O*-isopropylidene-6-deoxy-α-d-galacatopyranos-6-yl)glycinate (**3**) and *tert*-butyl-{2-[(1,7-dicarba-*closo*-dodecaboran-1-ylmethyl)-(1,2:3,4-di-*O*-isopropyliden-6-deoxy-α-d-galactopyranos-6-yl)amino]ethyl}carbamat (**4**) were obtained in moderate yields (54 and 51%, respectively), presumably due to steric hindrance (bulky carboranyl moiety and secondary amine derivative).

The final step was the deprotection of the acid-labile protecting groups (R(CO)O*^t^*Bu and RNH(CO)O*^t^*Bu, where R is the organic moiety) of compounds **3** and **4** with trifluoroacetic acid (TFA), with formation of the volatile reaction products isobutene and carbon dioxide leading to very pure products *N*-[(1,7-dicarba-*closo*-dodecaboran-1-yl)methyl]-*N*-(1,2:3,4-di-*O*-isopropylidene-6-deoxy-α-d-galacatopyranos-6-yl)glycine (**5**) and *N*^1^-(1,7-dicarba-*closo*-dodecaboran-1-yl-methyl)-*N*^1^-(1,2:3,4-di-*O*-isopropylidene-6-deoxy-α-d-galactopyranos-6-yl)ethane-1,2-diamine (**6**) in high yields due to a facile purification [61]. Anhydrous conditions must be employed to prevent cleavage of the monosaccharide protecting groups [61]. Compound **5** was first purified by column chromatography, which led to a noticeable loss of product (47% yield) due to the highly polar carboxylic acid group. Therefore, compounds **5** and **6** were purified by evaporation of all volatile reaction products with dichloromethane (DCM) as an entrainer, leading to pure compounds in good to excellent yields (65% and quantitative yield, respectively; details are given in the Supplementary Materials). Apparently, deprotection of the carboxylic acid ester (*tert*-butyl group) is less efficient than deprotection of the carbamate (Boc group).

The new compounds **1**–**6** were fully characterized by NMR spectroscopy (numbering scheme given in Figure S1), mass spectrometry and infrared spectroscopy and showed a purity of at least 95%. Furthermore, we were able to demonstrate that the same procedure can also be applied for the synthesis of the corresponding *ortho*-carborane derivative *N*^1^-[(1,2-dicarba-*closo*-dodecaboran-1-yl)methyl]-*N*^1^-(1,2:3,4-di-*O*-isopropylidene-6-deoxy-α-d-galactopyranos-6-yl)ethane-1,2-diamine (see the Supplementary Materials for further details).

**Figure 1:**
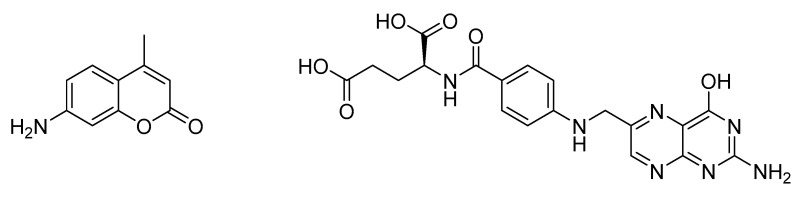
7-Amino-4-methylcoumarin (left) and folic acid (right) as two biomolecules selected to demonstrate the coupling behavior of glycine derivative **5** and ethylenediamine derivative **6**.

With the glycine derivative **5** and ethylenediamine derivative **6** in hand, exemplary coupling reactions with two selected biomolecules that play roles in cancer treatment were conducted. 7-Amino-4-methylcoumarin (Figure 1, left), already employed in the preparation of polyfunctional cancer therapeutics based on cisplatin derivatives [62] but not for the preparation of potential BNCT agents [63,64,65], was selected as a coupling partner for **5**. As coupling partner for amine **6**, folic acid (Figure 1, right) was chosen as this biomolecule has already been employed in the synthesis of potential BNCT agents [15,32,46,55,66]. Some cancer types overexpress folate receptors on their cell membrane surfaces, which can be used for selective uptake of the final BNCT agent in the respective cancer cells, increasing the efficacy of the therapy [32,67].

**Scheme 2:**
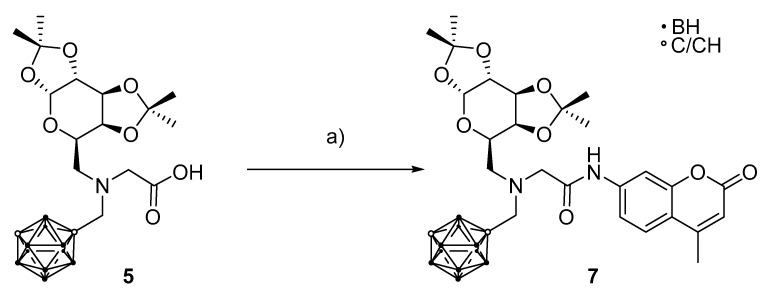
(**a**) 1.20 eq. 7-amino-4-methylcoumarin, 1.10 eq. POCl_3_, pyridine, 1 h, −18 °C, 27%.

The strategy for coupling glycine derivative **5** with the weakly nucleophilic coumarin derivative followed a procedure described by Quéléver and co-workers using phosphoryl chloride and pyridine [68] and resulted in *N^1^*-[(1,7-dicarba-*closo*-dodecaborane-1-yl)methyl]-*N^1^*-(1,2:3,4-di-*O*-isopropylidene-6-deoxy-α-d-galacatopyranos-6-yl)-*N^2^*-[(4-methyl)-2-oxo-2*H*-chromen-7-yl)glycineamide (**7**), albeit in low yield (27%) (Scheme 2). Attempts to use less harsh conditions (*N*,*N*’-dicyclohexylcarbodiimide (DCC) and *N*-hydroxysuccinimide (NHS)) for this coupling reaction were not successful [69]. Using different coupling reagents (1-ethyl-3-(3-dimethylaminopropyl)carbodiimide (EDCI) and hydroxybenzotriazol (HOBt) with *N,N*-diisopropylethylamine (DIPEA) as base [69]) yielded the desired product; however, it was in a very low yield of only 18%, indicating that this method is inferior to the phosphoryl chloride approach and the low reactivity of the coumarin derivative is the main issue. Compound **7** was fully characterized by NMR spectroscopy, mass spectrometry and infrared spectroscopy, proving the successful synthesis (with at least 95% purity) of this bioconjugate as a proof of principle in this approach.

**Scheme 3:**
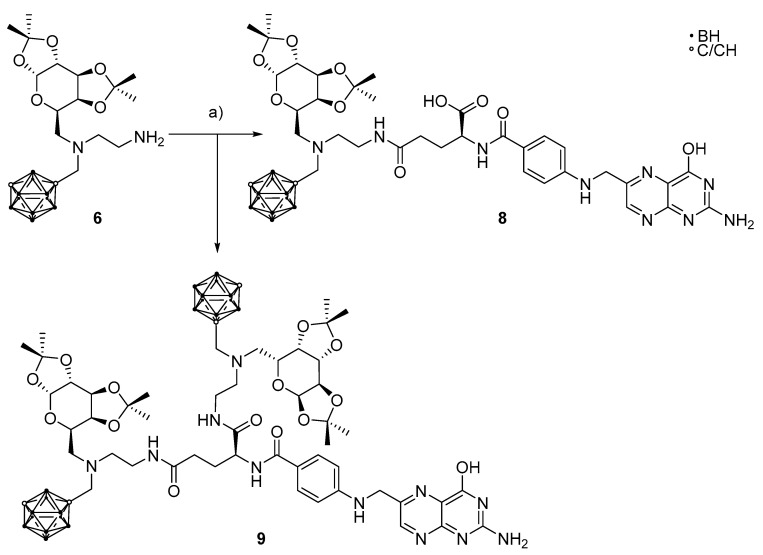
(**a**) 1.00 eq. folic acid, 1.00 eq. DCC, 1.00 eq. NHS, 1.10 eq. DIPEA, dimethylformamide, no yield determined.

The coupling reaction between folic acid and the primary amine **6** turned out to be more complicated due to the presence of two unprotected carboxylic acid groups in the former. Similar reactions have been reported where no additional protecting group was used for the secondary carboxylic acid group [55,66,70], as the primary COOH group exhibits a higher reactivity and, therefore, is more prone to undergo coupling reactions. However, here, the reaction of **6** with folic acid, following the procedure described by Trindade and co-workers, using DCC and NHS as activation reagents [70], gave both the desired monosubstituted species, namely *N*^2^-(4-{[(2-amino-4-hydroxypteridine-6-yl)methyl]amino}benzoyl)-*N*^5^-{2-[(1,7-dicarba-*closo*-dodecaboran-1-yl)methyl-(1,2:3,4-di-*O*-isopropyliden-6-deoxy-α-d-galactopyranos-6-yl)amino]ethyl}-l-glutamine (**8**) or its isomer, and the disubstituted species, namely (*S*)-2-(4-{[(2-amino-4-hydroxypteridine-6-yl)methyl]amino}benzamido)-*N*^1^,*N*^5^-bis-{2-[(1,7-dicarba-*closo*-dodecaboran-1-yl)methyl-(1,2:3,4-di-*O*-isopropylidene-6-deoxy-α-d-galactopyranos-6-yl)amino]ethyl}amino)pentanediamide (**9**) (Scheme 3), also verified by high-resolution mass spectrometry (*m*/*z* 883.5151 for **8** and *m*/*z* 1323.8829 for **9**). Obviously, in this case, the protocol from the literature [70] for conjugate formation with folic acid was not applicable, as we got a mixture of **8** and **9** in an unknown ratio. However, the obtained disubstituted folic acid derivative **9** has a high boron contents which could be useful for applications as a BNCT agent.

## 3. Materials and Methods

All reactions were carried out under nitrogen atmosphere using Schlenk techniques, if not reported otherwise. Anhydrous diethyl ether and DCM were obtained with an MBRAUN solvent purification system MB SPS-800 (M. Braun Inertgas-Systeme GmbH, Garching, Germany). MeCN and 2,4,6-collidine were dried over calcium hydride and distilled prior to use. Anhydrous tetrahydrofuran was dried over potassium and distilled prior to use. All solvents were stored over a molecular sieve (3 Å) under nitrogen atmosphere. 1,2-Dicarba-*closo*-dodecaborane(12) and 1,7-dicarba-*closo*-dodecaborane(12) are commercially available. 1-(Hydroxymethyl)-1,2-dicarba-*closo*-dodecaborane(12) [71], 1-(hydroxymethyl)-1,7-dicarba-*closo*-dodecaborane(12) [57], 1-(trifluoromethanesulfonylmethyl)-1,2-dicarba-*closo*-dodecaborane(12) [58], 1-(trifluoromethanesulfonylmethyl)-1,7-dicarba-*closo*-dodecaborane(12) [58], 1,2:3,4-di-*O*-isopropylidene-6-deoxy-α-d-galactopyranos-6-yltriflat [60] and 7-amino-4-methylcoumarin [72] were synthesized according to respective protocols from the literature. All other chemicals were commercially available and were used as received.

Thin-layer chromatography (TLC) with silica gel 60 F_254_ on glass, available from Merck KGaA (Darmstadt, Germany), or ALUGRAM^®^ XTRA SIL G/UV_254_ from Macherey-Nagel GmbH & Co. KG (Düren, Germany) on aluminum foil were used for monitoring the reactions. Carborane-containing spots were visualized with a 5% solution of PdCl_2_ in methanol. Non-carborane-containing spots were visualized with a basic potassium permanganate solution. For chromatography, silica gel (60 Å) with a particle diameter in the range of 0.035 to 0.070 mm was used. Prior to column chromatography, raw products were adsorbed on Celite^®^ S from Sigma-Aldrich Chemie GmbH (Taufkirchen, Germany).

NMR measurements were carried out on a Bruker AVANCE III HD spectrometer (Bruker Corporation, Billerica, MA, United States of America) with an Ascend^TM^ 400 magnet (Bruker Corporation, Billerica, MA, United States of America) at room temperature. Tetramethylsilane was used as internal standard for ^1^H- and ^13^C{^1^H}-NMR spectra; ^11^B- and ^11^B{^1^H}-NMR spectra were referenced to the Ξ scale [73]. NMR spectra were recorded at the following frequencies: ^1^H: 400.16 MHz, ^13^C: 100.63 MHz, ^11^B: 128.38 MHz. All chemical shifts are reported in parts per million (ppm). Assignment of the ^1^H and ^13^C signals was based on 2D-NMR spectra (H,H-COSY, H,C-HSQC, H,C-HMQC and H,C-HMBC). NMR data were interpreted with MestReNova [74]. NMR signals that appeared as broad overlapping signals with the shape of a multiplet or singlet in either ^1^H-, ^11^B{^1^H}- or ^11^B-NMR spectra were described as “br” (broad). The numbering scheme of the compounds for assignment of NMR signals is given in the Supplementary Materials (see Figure S1).

IR data were obtained with a PerkinElmer FT-IR spectrometer Spectrum 2000 (Perkin Elmer, Inc., Waltham, MA, United States of America) as KBr pellets and with a Thermo Scientific Nicolet iS5 with an ATR unit (Thermo Fisher Scientific, Waltham, MA, United States of America) in the range from 4000 to 400 cm^−1^.

High-resolution electrospray ionization mass spectrometry (ESI-HRMS) was performed with an ESI ESQUIRE 3000 PLUS spectrometer (Bruker Corporation, Billerica, MA, United States of America) with an IonTrap-analyzer from Bruker Daltonics or on a MicroTOF spectrometer from Bruker Daltonics with a ToF analyzer in negative or positive mode. As solvents for the measurements, DCM, MeCN, methanol or mixtures of these solvents were used. Interpretation of the spectra was carried out using MestReNova [74].

*Tert-Butyl-N-(1,2:3,4-di-O-isopropylidene-6-deoxy-α-d-galactopyranos-6-yl)glycinate* (**1**): Diisopropylethylamine (2.55 mL, 15.0 mmol, 2.96 eq.) was added dropwise under nitrogen atmosphere at room temperature to a solution of *tert*-butyl glycinate hydrochloride (1.12 g, 6.12 mmol, 1.20 eq.) and 1,2:3,4-di-*O*-isopropylidene-6-deoxy-α-d-galactopyranos-6-trifluoromethanesulfonate (2.00 g, 5.10 mmol, 1.00 eq.) in 50 mL MeCN. The reaction mixture was stirred for 72 h at 45 °C. The reaction was stopped by evaporating all volatile components under reduced pressure at 45 °C. The crude product was dissolved in ethyl acetate (100 mL) and washed with H_2_O and saturated NaCl solution. Subsequently, the aqueous layer was extracted with ethyl acetate (3 × 50 mL), dried with Na_2_SO_4_, filtered and the solvent was removed under reduced pressure. The crude material was purified by column chromatography using *n*-hexane/ethanol (14:1 (*R*_f_ = 0.41) to 10:1 (*R*_f_ = 0.58), (*v*/*v*)) as eluent. Compound **1** was obtained as colorless oil in 68% yield (1.30 g, 3.48 mmol). ^1^H-NMR (400 MHz, chloroform-*d*_1_): δ [ppm] = 1.33, 1.34, 1.45 and 1.54 (s, 12H, ^13, 13′, 14 and 14′^CH_3_), 1.46 (s, 9H, ^10, 10′ and 10′’^CH_3_), 2.77 to 2.92 (m, 2H, ^6^CH_2_), 3.26 to 3.40 (m, 2H, ^7^CH_2_), 3.88 (m, 1H, ^5^CH), 4.22 (dd, 1H, ^4^CH, *^3^J*_HH_ = 7.9 Hz, *^3^J*_HH_ = 4.9 Hz), 4.31 (dd, 1H, ^2^CH, *^3^J*_HH_ = 5.1 Hz, *^3^J*_HH_ = 2.4 Hz), 4.60 (dd, 1H, ^3^CH, *^3^J*_HH_ = 7.9 Hz, *^3^J*_HH_ = 4.9 Hz), 5.54 (d, 1H, ^1^CH, *^3^J*_HH_ = 5.1 Hz). ^13^C{^1^H}-NMR (100 MHz, chloroform-*d*_1_): δ [ppm] = 24.5 (s, ^13,13′,14 or 14′^CH_3_), 24.9 (s, ^13,13′,14 or 14′^CH_3_), 26.0 (s, ^13,13′,14 or 14′^CH_3_), 26.1 (s, ^13,13′,14 or 14′^CH_3_), 28.1 (s, ^10,10′ and 10′’^CH_3_), 49.1 (s, ^6^CH_2_), 51.8 (s, ^7^CH_2_), 67.1 (s, ^5^CH), 70.5 (s, ^2^CH), 70.8 (s, ^3^CH), 71.9 (s, ^4^CH), 80.9 (s, ^9^C_q_), 96.4 (s, ^1^CH), 108.4 (s, ^11^C_q_), 109.1 (s, ^12^C_q_), 171.4 (s, ^8^C_q_). IR (KBr): ṽ = 2982 (m, νCH-sp^3^), 2935 (m, νCH-sp^3^), 2361 (w), 1736 (s, νCO_Ester_), 1459 (w, δ_as._CH_2_), 1372 (m, δ_s._CH_3_), 1256 (m), 1213 (s), 1165 (s, ν_as._C-O-C_Ester_), 1114 (m), 1070 (s, νC-O-C_Ether_), 1003 (m), 918 (w), 898 (w), 854 (w), 804 (w), 771 (w), 650 (w), 512 (w) cm^−1^. ESI-HRMS: (*m*/*z*) calculated for [NaC_18_H_31_NO_7_]^+^ = 396.19986; observed 396.20153 [M+Na^+^]^+^; calculated for [C_18_H_32_NO_7_]^+^ = 374.21791; observed 374.21969 [M+H]^+^; calculated for [NaC_14_H_23_NO_7_]^+^ = 340.13726; observed 340.13844 [M-C_4_H_8_+Na]^+^; calculated for [C_14_H_24_NO_7_]^+^ = 318.15531; observed 318.15632 [M−C_4_H_8_+H]^+^.

*Tert-Butyl-{2-[(1,2:3,4-di-O-isopropylidene-6-deoxy-α-d-galactopyranos-6-yl)amino]-ethyl}carbamate* (**2**): A 250 mL round-bottom flask was charged with 3.20 g (8.16 mmol, 0.76 eq.) 1,2:3,4-di-*O*-isopropylidene-6-deoxy-α-d-galactopyranos-6-trifluormethanesulfonate and 60 mL MeCN. Then, 1.70 mL (1.73 g, 10.8 mmol, 1.00 eq.) *tert*-butyl-*N*-(2-aminoethyl)carbamate, dissolved in 10 mL MeCN, were added via a dropping funnel to this solution. The reaction mixture was cooled to 0 °C and, subsequently, 2.05 mL (1.56 g, 12.1 mmol, 1.12 eq.) *N*,*N*-diisopropylethylamine, dissolved in 10 mL MeCN, were added dropwise. After stirring for 15 min at 0 °C, the reaction mixture was warmed to 40 °C and stirred for two days. The reaction was stopped by cooling to room temperature and adding 30 mL saturated NH_4_Cl solution. All volatile components were removed under reduced pressure, and the remaining aqueous layer was extracted four times with 40 mL ethyl acetate, respectively. The combined organic layers were dried over MgSO_4_. The drying agent was filtered off and the solvent was removed under reduced pressure. The crude product was purified by column chromatography (a: DCM/methanol, 20:1, (*v*/*v*); b: acetone; c: *n*-hexane/isopropanol, 8:1 to 3:1, (*v*/*v*)). A total of 0.56 g *tert*-butyl-(2-{[bis(1,2:3,4-di-*O*-isopropylidene-6-deoxy-α-d-galactopyranos-6-yl)]amino}ethyl)-carbamate (**2′**) (*R*_f_ = 0.38, DCM/methanol, 20:1, (*v*/*v*), 0.87 mmol, 8%, analytical data are given in the Supplementary Materials) was obtained as a colorless solid, as well as 3.28 g *tert*-butyl-{2-[(1,2:3,4-di-*O*-isopropylidene-6-deoxy-α-d-galactopyranos-6-yl)amino]ethyl}carbamate (**2**) (*R*_f_ = 0.12 *n*-hexane/isopropanol, 5:1, (*v*/*v*), 8.27 mmol, 77%) as a colorless solid. ^1^H-NMR (400 MHz, acetone-*d*_6_): δ [ppm] = 1.34, 1.35, 1.43 and 1.52 (s, 21H, ^13,13′,13′’,16,16′,17 and 17′^CH_3_), 3.48 to 3.61 (m, 4H, ^8,9^CH_2_), 3.58 (m, 2H, ^6^CH_2_), 4.28 (td, 1H, ^5^CH, *^3^J*_HH_ = 6.0 Hz, *^3^J*_HH_ = 1.8 Hz), 4.39 (dd, 1H, ^4^CH, *^3^J*_HH_ = 7.9 Hz, *^3^J*_HH_ = 1.9 Hz), 4.45 (dd, 1H, ^2^CH, *^3^J*_HH_ = 5.0 Hz, *^3^J*_HH_ = 2.5 Hz), 4.73 (dd, 1H, ^3^CH, *^3^J*_HH_ = 7.8 Hz, *^3^J*_HH_ = 2.5 Hz), 5.56 (d, 1H, ^1^CH, *^3^J*_HH_ = 4.9 Hz), 6.64 (s, 1H, ^7^NH), 8.24 (s, 1H, ^10^NH). ^13^C{^1^H}-NMR (100 MHz, acetone-*d*_6_): δ [ppm] = 24.4, 25.0, 26.2, 28.5 (s, ^13, 13′, 13′’16,16′,17 and 17′^CH_3_), 38.2 (s, ^6^CH_2_), 49.3 (s, ^8 or 9^CH_2_), 50.7 (s, ^8 or 9^CH_2_), 64.6 (s, ^5^CH), 71.2 (s, ^2^CH), 71.6 (s, ^3^CH), 72.2 (s, ^4^CH), 80,6 (s, ^12^C_q_), 97.1 (s, ^1^CH) 109.9 and 110.7 (s, ^14, 15^C_q_). Carbonyl carbon atom ^11^C was not observed.

*Tert-Butyl-N-[(1,7-dicarba-closo-dodecaboran-1-yl)methyl]-N-(1,2:3,4-di-O-isopropylidene-6-deoxy-α-d-galacatopyranos-6-yl)glycinate* (**3**): A suspension of *tert*-butyl-*N*-(1,2:3,4-di-*O*-isopropylidene-6-deoxy-α-d-galactopyranos-6-yl)glycinate (**1**) (0.94 g, 2.52 mmol, 1.00 eq.), 1-(trifluoromethanesulfonylmethyl)-1,7-dicarba-*closo*-dodecaborane (0.93 g, 3.02 mmol, 1.20 eq.) and potassium carbonate (0.42 g, 3.02 mmol, 1.20 eq.) in 10 mL toluene was stirred at 95 °C for 43 h under nitrogen atmosphere. Then, the suspension was diluted with ethyl acetate and washed with 20 mL H_2_O and saturated NaCl solution. Subsequently, the aqueous layer was extracted with ethyl acetate (3 × 15 mL), the combined organic layers were dried over MgSO_4_ and filtered, and the solvent was removed under reduced pressure. The crude material was purified by column chromatography using *n*-hexane/ethyl acetate (20:1, (*v/v*), *R*_f_ = 0.50) and then *n*-hexane/ethanol (20:1, (*v/v*), *R*_f_ = 0.27) as eluent. Compound **3** was obtained as a colorless oil in 54% yield (721 mg, 1.36 mmol). ^1^H-NMR (400 MHz, chloroform-*d*_1_): δ [ppm] = 1.34, 1.42 and 1.56 (s, 12H, ^13,13′,14 and 14′^CH_3_), 1.44 (s, 9H, ^10,10′ and 10′’^CH_3_), 1.50 to 3.00 (br m, 10H, 10 BH), 2.83 to 2.91 (m, 1H, ^6^C*H*H), 2.89 (s, 1H, ^17^CH), 2.97 to 3.09 (dd, 1H, ^6^CH*H*, ^2^*J*_HH_ = 14.1 Hz, ^3^*J*_HH_ = 7.0 Hz), 3.13 (d, 1H, ^15^C*H*H, ^2^*J*_HH_ = 15.7 Hz), 3.23 (d, 1H, ^15^CH*H*, ^3^*J*_HH_ = 15.7 Hz), 3.36 (d, 1H, ^7^C*H*H, ^3^*J*_HH_ = 17.8 Hz), 3.53 (d, 1H, ^7^CH*H*, ^3^*J*_HH_ = 17.8 Hz), 3.84 (m, 1H, ^5^CH), 4.25 (dd, 1H, ^4^CH, ^3^*J*_HH_ = 7.9 Hz, ^3^*J*_HH_ = 1.9 Hz), 4.29 (dd, 1H, ^2^CH, ^3^*J*_HH_ = 5.1 Hz, ^3^*J*_HH_ = 2.4 Hz), 4.60 (dd, 1H, ^3^CH, ^3^*J*_HH_ = 7.9 Hz, ^3^*J*_HH_ = 2.4 Hz), 5.50 (d, 1H, ^1^CH, ^3^*J*_HH_ = 5.1 Hz). ^13^C{^1^H}-NMR (100 MHz, chloroform-*d*_1_): δ [ppm] = 24.6, 24.9, 26.0 (s, ^13,13′,14 and 14′^CH_3_), 28.2 (s, ^10,10′ and 10′’^CH_3_), 54.8 (s, ^17^CH), 55.0 (s, ^6^CH_2_), 55.9 (s, ^7^CH_2_), 60.9 (s, ^15^CH_2_), 66.8 (s, ^5^CH), 70.4 (s, ^2^CH), 70.8 (s, ^3^CH), 71.9 (s, ^4^CH), 78.5 (s, ^16^C_q_), 81.5 (s, ^9^C_q_), 96.5 (s, ^1^CH), 108.5 and 109.1 (s, ^11, 12^C_q_), 170.8 (s, ^8^C_q_). ^11^B{^1^H}-NMR (128 MHz, chloroform-*d*_1_): δ [ppm] = −15.7 (s, 2B), −13.6 (s, 2B), −10.9 (s, 4B), −9.5 (s, 1B), −4.4 (s, 1B). ESI-HRMS: (*m*/*z*) calculated for [NaC_21_H_43_B_10_NO_7_]^+^ = 552.3941; observed 552.3966 [M+Na]^+^.

*Tert-Butyl-{2-[(1,7-dicarba-closo-dodecaboran-1-yl)methyl-(1,2:3,4-di-O-isopropyliden-6-deoxy-α-d-galactopyranos-6-yl)amino]ethyl}carbamat* (**4**): A total of 1.66 g (4.12 mmol, 1.00 eq.) *tert*-butyl-{2-[(1,2:3,4-di-*O*-isopropylidene-6-deoxy-α-d-galactopyranos-6-yl)amino]-ethyl}carbamate (**2**) was placed in a 50 mL round-bottom flask and dissolved in 10 mL toluene. Subsequently, 1.51 g (4.94 mmol, 1.20 eq.) 1-(trifluoromethanesulfonylmethyl)-1,7-dicarba-*closo*-dodecaborane(12), dissolved in 10 mL toluene, and 0.68 g (4.94 mmol, 1.20 eq.) K_2_CO_3_ were added. The suspension was heated to 98 °C and stirred for 48 h. The reaction was stopped by adding 20 mL distilled water and 4 mL saturated NaCl solution. The aqueous layer and organic layer were separated. The aqueous layer was extracted two times with 20 mL ethyl acetate. The combined organic layers were dried over MgSO_4_, the drying agent was filtered off and the solvent was removed under reduced pressure. The resulting yellow-brownish oil was purified by column chromatography (n-hexane/ethyl acetate, 5:1, (*v/v*)) and **4** (1.17 g, 2.09 mmol, 51%, *R*_f_ = 0.24 (n-hexane/ethyl acetate, 5:1, (*v/v*))) was isolated as a colorless foamy solid. ^1^H-NMR (400 MHz, chloroform-*d*_1_): δ [ppm] = 1.19 to 3.41 (m, br, 10H, 10 BH), 1.34, 1.36, 1.45 and 1.56 (s, 12H, ^15,15′,16 and 16′^CH_3_), 1.45 (s, 9H, ^12, 12′ and 12′’^CH_3_), 2.55 to 2.63 (m, 1H, ^7 or 8^C*H*H), 2.63 to 2.72 (m, 1H, ^6^C*H*H), 2.80 to 2.88 (m, 2H, ^7 or 8^CH*H* and ^6^C*H*H), 2.90 (s, 1H, ^19^CH), 2.94 to 3.07 (m, 2H, ^17^C*H*H and ^17^CH*H*), 3.10 to 3.25 (m, 2H, ^7 and 8^CH*H*), 3.85 (m, 1H, ^5^CH), 4.24 (d, 1H, ^4^CH, ^3^*J*_HH_ = 7.9 Hz), 4.31 (dd, 1H, ^2^CH, ^3^*J*_HH_ = 5.1 Hz, ^3^*J*_HH_ = 2.4 Hz), 4.62 (dd, 1H, ^3^CH, ^3^*J*_HH_ = 7.9 Hz, ^3^*J*_HH_ = 2.4 Hz), 5.35 (s, br, 1H, ^9^NH), 5.51 (d, 1H, ^1^CH, ^3^*J*_HH_ = 5.1 Hz). ^13^C{^1^H}-NMR (100 MHz, chloroform-*d*_1_): δ [ppm] = 24.4, 24.9, 26.0 (s, ^15,15′,16 and 16′^CH_3_), 28.5 (s, ^12,12′ and 12′’^CH_3_), 38.4 (s, ^7 or 8^CH_2_), 53.0 (s, ^6^CH_2_), 54.6 (s, ^7 or 8^CH_2_) 54.7 (s, ^19^CH), 60.7 (s, ^17^CH_2_), 65.8 (s, ^5^CH), 70.4 (s, ^2^CH), 70.9 (s, ^3^CH), 71.8 (s, ^4^CH), 77.8 (s, ^18^C_q_), 79.0 (s, ^11^C_q_), 96.5 (s, ^1^CH), 108.4 and 109.3 (s, ^13 and 14^C_q_), 156.1 (s, ^10^C_q_). ^11^B{^1^H}-NMR (128 MHz, chloroform-*d*_1_): δ [ppm] = −15.6 (s, 2B), −13.6 (s, 2B), −10.8 (s, 4B), −9.3 (s, 1B), −4.3 (s, 1B). IR (KBr): ṽ = 3390 (w, νNH), 2979 (w, νCH-sp^3^), 2933 (w, νCH-sp^3^), 2594 (m, νBH), 1707 (m, amide I), 1504 (m, amide II), 1455 (m, δCH), 1366 (m, δCH), 1252 (m, amide III) cm^−1^. ESI-HRMS: (*m*/*z*) calculated for [C_22_H_47_B_10_N_2_O_7_]^+^ = 559.4386; observed 559.4395 [M+H]^+^; calculated for [NaC_22_H_46_B_10_N_2_O_7_]^+^ = 581.4282; observed 581.4208 [M+Na]^+^.

*N-[(1,7-dicarba-closo-dodecaborane-1-yl)methyl]-N-(1,2:3,4-di-O-isopropylidene-6-deoxy-α-d-galacatopyranos-6-yl)glycine* (**5**): Method A. Anhydrous TFA (4.20 mL, 54.4 mmol, 40.0 eq.) was added dropwise under nitrogen atmosphere at 0 °C to a solution of *tert*-butyl-*N*-[(1,7-dicarba-*closo*-dodecaboran-1-yl)methyl]-*N*-(1,2:3,4-di-*O*-isopropylidene-6-deoxy-α-d-galacatopyranos-6-yl)glycinate (**3**) (0.71 g, 1.36 mmol, 1.00 eq.) in 4.20 mL DCM. The mixture was warmed to room temperature and stirred for 3 h. When the reaction was completed, TFA and DCM were removed under reduced pressure. DCM and diethyl ether were used as an entrainer to remove remaining TFA. The crude material was purified by column chromatography using *n*-hexane/ethyl acetate (10:1, (*v*/*v*)) as eluent. Diethyl ether was used to remove remaining solvent. Compound **5** was obtained as a colorless solid in 47% yield (300 mg, 0.63 mmol).

Method B. A Schlenk flask was charged with *tert*-butyl-*N*-[(1,7-dicarba-*closo*-dodecaboran-1-yl)methyl]-*N*-(1,2:3,4-di-*O*-isopropylidene-6-deoxy-α-d-galacatopyranos-6-yl)-glycinate (**3**) (0.50 g, 0.94 mmol, 1.00 eq.). Then, 4.0 mL anhydrous TFA (52.2 mmol, 55.6 eq.) were added and the resulting solution was stirred at room temperature for 3 h. Afterwards, about 4 mL DCM were added and both, TFA and DCM, were removed under reduced pressure. This process was repeated three more times with DCM as an entrainer to remove remaining TFA. Then, 3 mL concentrated NaHCO_3_ solution were added to the obtained crude product while stirring and the mixture was subsequently sonicated for 15 min, resulting in a thick, cloudy white suspension with a brownish oil-like layer on top. Upon addition of 2 mL DCM, the oil-like layer was dissolved and gas evolution was observed. Once the gas evolution had almost stopped, the solution was stirred for five more minutes. Subsequently, the aqueous layer was removed using a syringe and extracted two times with 2 mL ethyl acetate each. The combined organic layers were washed twice with 2 mL of water. Afterwards, the combined organic layers were dried over MgSO_4_ and filtered. Finally, the solvent was removed under reduced pressure, affording compound **5** as a yellowish foamy solid in 65% yield (290 mg, 0.612 mmol). ^1^H-NMR (400 MHz, chloroform-*d*_1_): δ [ppm] = 1.33, 1.34, 1.43 and 1.56 (s, 12H, ^11,11′,12 and 12′^CH_3_), 1.50 to 3.00 (m, br, 10H, 10 BH), 2.91 to 2.99 (m, 3H, ^6^CH_2_ and ^15^CH), 3.17 (d, 1H, ^13^C*H*H, ^2^*J*_HH_ = 20.0 Hz), 3.21 (d, 1H, ^13^CH*H*, ^2^*J*_HH_ = 20.0 Hz), 3.48 (d, 1H, ^7^C*H*H, ^2^*J*_HH_ = 18.2 Hz), 3.59 (d, 1H, ^7^CH*H*, ^2^*J*_HH_ = 18.2 Hz), 3.87 (m, 1H, ^5^CH), 4.15 (dd, 1H, ^4^CH, *^3^J*_HH_ = 7.9 Hz, *^3^J*_HH_ = 2.0 Hz), 4.35 (dd, 1H, ^2^CH, *^3^J*_HH_ = 5.2 Hz, *^3^J*_HH_ = 2,4 Hz), 4.62 (dd, 1H, ^3^CH, *^3^J*_HH_ = 7.9 Hz, *^3^J*_HH_ = 2.4 Hz), 5.55 (d, 1H, ^1^CH, *^3^J*_HH_ = 5.1 Hz). **^1^**^3^C{^1^H}-NMR (100 MHz, chloroform-*d*_1_): δ [ppm] = 24.4, 24.8, 25.95 and 26.03 (s, ^11,11′,12 and 12′^CH_3_), 54.6 (s, ^6^CH_2_), 55.3 (s, ^15^CH), 57.2 (s, ^7^CH_2_), 59.1 (s, ^13^CH_2_), 65.5 (s, ^5^CH), 70.4 (s, ^2^CH), 70.8 (s, ^3^CH), 71.6 (s, ^4^CH), 76.3 (s, ^14^C_q_), 96.4 (s, ^1^CH), 108.8 and 109.7 (s, ^9 and 10^C_q_), 172.4 (s, ^8^C_q_). ^11^B{^1^H}-NMR (128 MHz, chloroform-*d*_1_): δ [ppm] = −15.7 (s, 2B), −13.5 (s, 2B), −11.3 (s, 2B), −10.6 (s, 2B), −9.0 (s, 1B), −4.5 (s, 1B). IR (KBr): ṽ = 3061 (w, νCH_2_-sp^2^), 2985 (w, ν_as._CH_2_-sp^3^), 2961 (w, ν_s._CH_2_-sp^3^), 2931 (m, ν_as._CH_3_-sp^3^), 2871 (w, ν_s._CH_3_-sp^3^), 2595 (s, νBH-sp^3^), 1716 (m, νC=O_carboxylic acid_), 1456 (m, δ_as._CH_3_-sp^3^), 13 (m, δ_s._CH_3_-sp^3^), 1066 (s, νC-O-C_ether_) cm^-1^. ESI-HRMS: (*m*/*z*) calculated for [C_17_H_36_B_10_NO_7_]^+^ = 474.3495; observed 474.3485 [M+H]^+^.

*N^1^-[(1,7-Dicarba-closo-dodecaborane-1-yl)methyl]-N^1^-(1,2:3,4-di-O-isopropylidene-6-deoxy-α-d-galactopyranos-6-yl)ethane-1,2-diamine* (**6**): A 25 mL round-bottom flask was charged with 0.30 g (0.54 mmol, 1.00 eq.) tert-butyl-{2-[(1,7-dicarba-*closo*-dodecaboran-1-yl)methyl-(1,2,:3,4-di-*O*-isopropylidene-6-deoxy-α-d-galactopyranos-6-yl)amino]ethyl}-carbamate (**4**) and 2.00 mL (26.0 mmol, 48.2 eq.) TFA were added. The reaction mixture was stirred for 3 h at room temperature. The reaction was stopped by adding 4 mL DCM with subsequent evaporation of all volatile components under reduced pressure. This procedure was repeated three times. The resulting crude product was further purified by adding 4 mL saturated NaHCO_3_ solution and sonication for about 15 min. Again, 3 mL DCM were added with stirring, under observation of gas evolution, and after 5 min the resulting layers were separated. The aqueous layer was extracted with 3 mL ethyl acetate. The combined organic layers were washed twice with 2 mL distilled water each. The organic layer was dried over MgSO_4_, the drying agents were filtered off and the solvent was removed under reduced pressure. Compound **6** (0.25 g, 0.54 mmol, quant., *R*_f_ = 0.03, n-hexane/ethyl acetate, 5:1, (*v/v*)) was isolated as a colorless foamy solid. ^1^H-NMR (400 MHz, chloroform-*d*_1_): δ [ppm] = 1.33, 1.34, 1.45, 1.54 (s, 12H, ^12,12′,13 and 13′^CH_3_), 1.40 to 3.16 (m, br, 10H, 10 BH), 2.62 to 2.69 (m, 1H, ^7 or 8^C*H*H), 2.72 to 2.77 (m, 2H, ^7 or/and 8^CH*H*), 2.77 to 2.86 (m, 3H, ^7 or 8^CH*H* and ^6^CH_2_), 2.92 (s, 1H, ^16^CH), 3.00 (d, 1H, ^14^C*H*H, ^2^*J*_HH_ = 15.4 Hz), 3.11 (d, 1H, ^14^CH*H*, ^2^*J*_HH_ = 15.4 Hz) 3.87 (m, 1H, ^5^CH), 4.17 (dd, 1H, ^4^CH, ^3^*J*_HH_ = 7.9 Hz, ^3^*J*_HH_ = 1.9 Hz), 4.30 (dd, 1H, ^2^CH, ^3^*J*_HH_ = 5,2 Hz, ^3^*J*_HH_ = 2.4 Hz), 4.60 (dd, 1H, ^3^CH, ^3^*J*_HH_ = 7.9 Hz, ^3^*J*_HH_ = 2.4 Hz), 5.52 (d, 1H, ^1^CH, ^3^*J*_HH_ = 5.1 Hz). ^13^C{^1^H}-NMR (100 MHz, chloroform-*d*_1_): δ [ppm] = 24.5, 24.8, 25.96 and 26.0 (s, ^12,12′,13 and 13′^CH_3_), 39.8 (s, ^7 or 8^CH_2_), 53.2 (s, ^6^CH_2_), 55.0 (s, ^16^CH), 57.7 (s, ^7 or 8^CH_2_), 60.2 (s, ^14^CH_2_), 66.1 (s, ^5^CH), 70.3 (s, ^2^CH), 70.8 (s, ^3^CH), 72.0 (s, ^4^CH), 96.5 (s, ^1^CH), 108.5 and 109.3 (s, ^10 and 11^C_q_), ^15^C_q_ was not observable (assumed at 77.7 ppm). ^11^B{^1^H}-NMR (128 MHz, chloroform-*d*_1_): δ [ppm] = −15.6 (s, 2B), −13.5 (s, 2B), −11.2 (s, 2B), −10.8 (s, 2B), −9.3 (s, 1B), −4.3 (s, 1B). IR (KBr): ṽ = 2986 (w, νCH-sp^3^), 2933 (w, νCH-sp^3^), 2593 (m, νBH-sp^3^), 1685 (w, δNH-sp^3^), 1455 (w, δCH-sp^3^), 1381 (m, δCH-sp^3^) cm^−1^. ESI-HRMS: calculated for [C_17_H_39_B_10_N_2_O_5_]^+^ = 459.3862; observed 459.3851 [M+H]^+^; calculated for [C_34_H_77_B_20_N_4_O_10_]^+^ = 918.7724; observed 918.7607 [2M+H]^+^.

*N^1^-[(1,7-dicarba-closo-dodecaboran-1-yl)methyl]-N^1^-(1,2:3,4-di-O-isopropylidene-6-deoxy-α-d-galacatopyranos-6-yl)-N^2^-[(4-methyl)-2-oxo-2H-chromen-7-yl)glycineamide* (**7**): Method A. Under nitrogen atmosphere, 1 mL (0.98 g, 12.4 mmol) of pyridine was added to a mixture of N-[(1,7-dicarba-*closo*-dodecaboran-1-yl)methyl]-*N*-(1,2:3,4-di-*O*-isopropylidene-6-deoxy-α-d-galacatopyranos-6-yl)glycine (**5**) (100 mg, 0.21 mmol, 1.00 eq.) and 7-amino-4-methylcoumarin (44.4 mg, 0.25 mmol, 1.20 eq.). After degassing the yellowish solution with nitrogen, phosphorus(V) oxychloride (20.0 µL, 0.23 mmol, 1.10 eq.) was added dropwise at −18 °C. The solution immediately changed to a red suspension and after a while back to a yellow solution. The mixture was stirred for 1 h at −18 °C. After the reaction was finished, the mixture was poured into H_2_O and extracted with ethyl acetate. Subsequently, the combined organic layers were washed with a NaHCO_3_ solution and a saturated NaCl solution and then dried over MgSO_4_. After filtration, the solvent was removed under reduced pressure and the crude product was purified by column chromatography using n-hexane/ethyl acetate (1:1, (*v/v*), *R*_f_ = 0.51) as eluent. Compound **7** was obtained as a brownish solid in 27% yield (36 mg, 5.71 µmol).

Method B. *N*-[(1,7-dicarba-*closo*-dodecaboran-1-yl)methyl]-*N*-(1,2:3,4-di-*O*-isopropylidene-6-deoxy-α-d-galacatopyranos-6-yl)glycine (86.1 mg, 0.18 mmol, 1.00 eq.) was added to a Schlenk tube and was dissolved in 5 mL absolute DCM. Subsequently, the solution was cooled to 0 °C in an ice bath and 7-amino-4-methylcoumarin (39.1 mg, 0.22 mmol, 1.23 eq.), HOBt (29.5 mg, 0.22 mmol, 1.20 eq.), EDCI (45.1 mg, 0.24 mmol, 1.30 eq.) and DIPEA (80 µL, 0.46 mmol, 2.54 eq.) were added. Afterwards, the ice bath was removed, the reaction mixture was allowed to reach room temperature and stirred at room temperature for 22 h. Subsequently, the solvent was removed under reduced pressure and the residue was redissolved in 20 mL ethyl acetate and washed with 30 mL of 1 N HCl, concentrated NaHCO_3_ and NaCl solution, respectively. The organic layer was dried over Na_2_SO_4_, filtered and the solvent was removed under reduced pressure. The obtained crude product was purified by column chromatography using *n*-hexane/ethyl acetate (1:1 (*R*_f_ = 0.51) to 1:2, (*v*/*v*)) as eluent, affording compound **7** as a sticky, brownish solid in 18% yield (20 mg, 32 µmol). ^1^H-NMR (400 MHz, chloroform-*d*_1_): δ [ppm] = 1.28, 1.29, 1.38, 1.59 (s, 12H, ^12,12′,13 and 13′^CH_3_), 1.50 and 3.00 (m, br, 10H, 10 BH), 2.42 (d, 3H, ^23^CH_3_, *^4^J*_HH_ = 1.2 Hz), 2.86 to 2.97 (m, 3H, ^6^C*H*H, ^6^CH*H* and ^16^CH), 3.16 (d, 1H, ^14^C*H*H, ^2^*J*_HH_ = 15.5 Hz), 3.25 (d, 1H, ^14^CH*H*, ^2^*J*_HH_ = 15.5 Hz), 3.43 (d, 1H, ^7^C*H*H, ^2^*J*_HH_ = 17.5 Hz), 3.52 (d, 1H, ^7^CH*H*, ^2^*J*_HH_ = 17.4 Hz), 3.93 (m, 1H, ^5^CH, *J*_HH_ = 9.7 and 2.5 Hz), 4.10 (dd, 1H, ^4^CH, ^3^*J*_HH_ = 7.8 Hz, ^3^*J*_HH_ = 2.0 Hz), 4.41 (dd, 1H, ^2^CH, *^3^J*_HH_ = 5.2 Hz, *^3^J*_HH_ = 2.4 Hz), 4.63 (dd, 1H, ^3^CH, *^3^J*_HH_ = 7.8 Hz, *^3^J*_HH_ = 2.4 Hz), 5.69 (d, 1H, ^1^CH, *^3^J*_HH_ = 5.2 Hz), 6.21 (d, 1H, ^21^CH, ^4^*J*_HH_ = 1.4 Hz), 7.53 to 7.58 (m, 2H, ^18^CH and ^25^CH), 7.71 (dd, 1H, ^26^CH, ^3^*J*_HH_ = 8.7 Hz, ^4^*J*_HH_ = 2.0 Hz), 9.57 (s, 1H, ^9^NH). ^13^C{^1^H}-NMR (100 MHz, chloroform-*d*_1_): δ [ppm] = 18.6 (s, ^23^CH_3_), 24.4, 24.7, 25.9 and 26.1 (s, ^12,12′,13 or 13′^CH_3_), 54.5 (s, ^6^CH_2_), 55.3 (s, ^16^CH), 58.7 (s, ^14^CH_2_), 60.0 (s, ^7^CH_2_), 64.8 (s, ^5^CH), 70.3 (s, ^2^CH), 70.8 (s, ^3^CH), 71.7 (s, ^4^CH), 75.9 (s, ^15^C_q_), 96.5 (s, ^1^CH), 107.0 (s, ^18^CH), 108.9 and 109.6 (s, ^10 and 11^C_q_), 113.4 (s, ^21^CH), 115.7 (s, ^26^CH), 116.2 (s, ^22^C_q_), 125.2 (s, ^25^CH), 141.2 (s, ^17^C_q_), 152.3 (s, ^24^C_q_), 154.3 (s, ^19^C_q_), 161.2 (s, ^20^C_q_), 169.3 (s, ^8^C_q_). ^11^B{^1^H}-NMR (128 MHz, chloroform-**d*_1_*): δ [ppm] = −15.6 (s, 2B), −13.4 (s, 2B), −11.2 (s, 2B), −10.5 (s, 2B), −8.9 (s, 1B), −4.4 (s, 1B). IR (KBr): ṽ = 3288 (m, ν(NH, secondary amide (trans)), 3058 (w, νCH-sp^2^), 2985 (w, ν_as_CH_2_-sp^3^), 2957 (w, ν_s_CH_2_-sp^3^), 2922 (m, ν_as_CH_3_-sp^3^), 2852 (m, ν_s_CH_3_-sp^3^), 2595 (m, νBH-sp^3^), 1727 (s, νC=O_lactone_), 1706 (s, vC=O_amide_, amide I), 1616 (νC=C, α,ß-unsaturation), 1567 (νC=C, aromatic or δNH, amide II), 1519 (vC=C, aromatic or νCN, amide II), 1454 (m, δ_as_CH_3_-sp^3^), 1369 (m, δ_s_CH_3_-sp^3^), 1066 (s, νC-O-C_Ether_), 901 (m, δCH-sp^2^/aromatic), 853, 806 (m, δCH-sp^2^/aromatic) cm^−1^. ESI-HRMS: (*m*/*z*) calculated for [NaC_27_H_42_B_10_N_2_O_8_]^+^ = 653.3843; observed 653.3840 [M+Na]^+^.

*N*^2^-(4-{[(2-Amino-4-hydroxypteridine-6-yl)methyl]amino}benzoyl)-*N*^5^-{2-[(1,7-dicarba-*closo*-dodecaboran-1-ylmethyl)-(1,2:3,4-di-*O*-isopropyliden-6-deoxy-α-d-galactopyranos-6-yl)amino]ethyl}-l-glutamine (**8**) and (*S*)-2-(4-{[(2-amino-4-hydroxypteridine-6-yl)methyl]amino}benzamido)-*N^1^*,*N^5^*-bis-{2-[(1,7-dicarba-*closo*-dodecaborane-1-yl)methyl-(1,2:3,4-di-*O*-isopropylidene-6-deoxy-α-d-galactopyranos-6-yl)amino]ethyl}amino)pentanediamide (**9**): A 100 mL Schlenk flask was charged with 0.24 g (0.55 mmol, 1.00 eq.) folic acid and 20 mL dimethylformamide were added. The mixture was sonicated for 15 min and, subsequently, warmed to 37 °C for 15 min until a clear solution was obtained. To this mixture, 0.11 g (0.55 mmol, 1.00 eq.) DCC and 0.06 g (0.55 mmol, 1.00 eq.) NHS were added. The reaction mixture was stirred for 16 h at room temperature. Afterwards, 0.25 g (0.55 mmol, 1.00 eq.) *N*^1^-[(1,7-dicarba-*closo*-dodecaborane-1-yl)methyl]-*N*^1^-(1,2:3,4-di-*O*-isopropylidene-6-deoxy-α-d-galactopyranos-6-yl)ethane-1,2-diamine (**6**), dissolved in 7 mL dimethylformamide, were added to this solution and the mixture was stirred overnight at room temperature. The resulting suspension was filtered under inert conditions. Subsequently, 0.10 mL (0.61 mmol, 1.10 eq.) *N*,*N*-diisopropylethylamine were added and the mixture was stirred overnight at room temperature. The reaction was stopped by adding 50 mL ice-cold diethyl ether. Simultaneously, the mixture was cooled in an ice bath. Completion of the precipitation was achieved by storage for one additional night at −20 °C. The resulting precipitate was filtered off and washed with 10 mL ice-cold diethyl ether. The precipitate was suspended in 5 mL diethyl ether and subsequently sonicated for 15 min. Afterwards, the raw product was filtered again. The orange-red solid was washed with 5 mL diethyl ether and the previously described procedure was repeated one more time. Afterwards, the precipitate was dried in vacuo. It was not possible to isolate the desired compound **8**, but mass spectrometry revealed the presence of *N*^2^-(4-{[(2-amino-4-hydroxypteridine-6-yl)methyl]amino}benzoyl)-*N*^5^-{2-[(1,7-dicarba-*closo*-dodecaboran-1-yl)methyl-(1,2:3,4-di-*O*-isopropyliden-6-deoxy-α-d-galactopyranos-6-yl)amino]ethyl}-l-glutamine (**8**) and (*S*)-2-(4-{[(2-amino-4-hydroxypteridine-6-yl)methyl]amino}benzamido)-*N^1^*,*N^5^*-bis-{2-[(1,7-dicarba-*closo*-dodecaborane-1-yl)methyl-(1,2:3,4-di-*O*-isopropylidene-6-deoxy-α-d-galactopyranos-6-yl)amino]ethyl}amino)pentanediamide (**9**). Due to impurities, it was not possible to determine a yield based on HRMS. **8**: ESI-HRMS: (*m*/*z*) calculated for [C_36_H_56_B_10_N_9_O_10_]^+^ = 883.5139; observed 883.5151 [M+H]^+^; calculated for [NaC_36_H_55_B_10_N_9_O_10_]^+^ = 905.4959; observed 905.4963 [M+Na]^+^; calculated for [KC_36_H_55_B_10_N_9_O_10_]^+^ = 921.4700; observed 921.4715 [M+K]^+^. **9**:1E7D IR (KBr): ṽ = 3315 (w, νNH-sp^3^), 2931 (w, νCH-sp^3^), 2850 (w, νCH-sp^3^), 2594 (w, νBH-sp^3^), 1723 (m, amide I), 1687 (m, amide II), 1605 (s, νC=C_arom._), 1412 (m, δCH-sp^3^) cm^−1^. ESI-HRMS: (*m*/*z*) calculated for [C_53_H_92_B_20_N_11_O_14_]^+^ = 1323.8830; observed 1323.8829 [M+H]^+^; calculated for [NaC_53_H_91_B_20_N_11_O_14_]^+^ = 1345.8649; observed 1345.8625 [M+Na]^+^; calculated for [KC_53_H_91_B_20_N_11_O_14_]^+^ = 1361.8390; observed 1361.8349 [M+K]^+^.

## 4. Conclusions

In this work we reported the successful design of a novel modular, small-molecule-based approach to synthesizing boron-rich compounds bearing a carboxylic acid group or a primary amine group as potential coupling partners for suitable tumor-selective biomolecules. As proof of concept, conjugates with 7-amino-4-methylcoumarin and folic acid were obtained. While the present work focused on the development of a synthetic protocol, the next steps will include the deprotection of the respective galactopyranosyl protecting groups under acidic aqueous conditions [61] followed by biological investigations.

## Data Availability

The data presented in this study are available on request from the corresponding author.

## Supplementary Materials

Supplementary information is available online, including the numbering scheme of the isolated compounds **1**‒**7**, NMR spectra of compounds **5**, **6** and **7** and mass spectra of **8** and **9**, additional synthetic procedures and analytical data for **2′**, **ESI-3**, **ESI-3′**, **ESI-4** and 1-(trifluoromethanesulfonylmethyl)-1,7-dicarba-*closo*-dodecaborane(12), crystallographic information for compound **ESI-3′**, information about the exploration of the optimization for the synthesis of **3** and the deprotection protocol for **4,** and the extension of the synthetic protocol to *ortho*-carborane derivatives.

## Sample Availability

Samples of the compounds are not available from the authors.
