# Supplementary material for: Modular Synthetic Approach to Carboranyl‒Biomolecules Conjugates
Source: Molecules. 2021 Apr 3;26(7):2057. doi: 10.3390/molecules26072057 (PMC8038343; doi:10.3390/molecules26072057)
Supplement: Supplementary file 1 [file molecules-26-02057-s001.pdf]

Electronic Supplementary Information

Modular Synthetic Approach to Carboranyl–Biomolecules Conjugates

Dr. Martin Kellert[1], B.Sc. Jan-Simon Jeshua Friedrichs[1], B.Sc. Nadine Anke Ullrich[1], B.Sc. Alexander Feinhals[1], B.Sc. Jonas Tepper[1], Dr. Peter Lönnecke[1], Prof. Dr. Dr. h.c. Evamarie Hey-Hawkins[1,*]

[1] Leipzig University, Faculty of Chemistry and Mineralogy, Institute of Inorganic Chemistry, Johannisallee 29, 04103 Leipzig, Germany

***** Correspondence: hey@uni-leipzig.de; Tel.: +49-341-97-36151 (E. H.-H.)

Contents | Page

Contents	Page
1. Numbering scheme of compounds **1** to **7**	3
2. Depiction of the NMR spectra of compounds **5**, **6**, **7** and mass spectrum of **8** and **9**	4
3. Optimization of reaction conditions for the synthesis of **3**	10
4. Optimization of the deprotection protocol for **4**	11
5. Side product *tert*-butyl-(2-{[bis(1,2:3,4-di-*O*-isopropylidene-6-deoxy-α-D-galactopyranos-6-yl)]-amino}ethyl)carbamate (**2’**)	13
6. Extension of the synthetic protocol to *ortho*-carborane derivatives	14
6.1. Synthetic procedure and analytical data of 1-(trifluoromethane-sulfonylmethyl)-1,7-dicarba-*closo*-dodecaborane(12)	19
6.2. Synthetic procedures and analytical data of *tert*-butyl-{2-[(1,2-dicarba-*closo*-dodecaborane-1-yl)methyl]aminoethyl}-carbamate (**ESI-3**) and *N*’-[(1,2-dicarba-closo-dodecaborane-1-yl)-methyl]ethane-1,2-diamine (**ESI-4**)	20
7. References	23

1. Numbering scheme of compounds 1 to 7

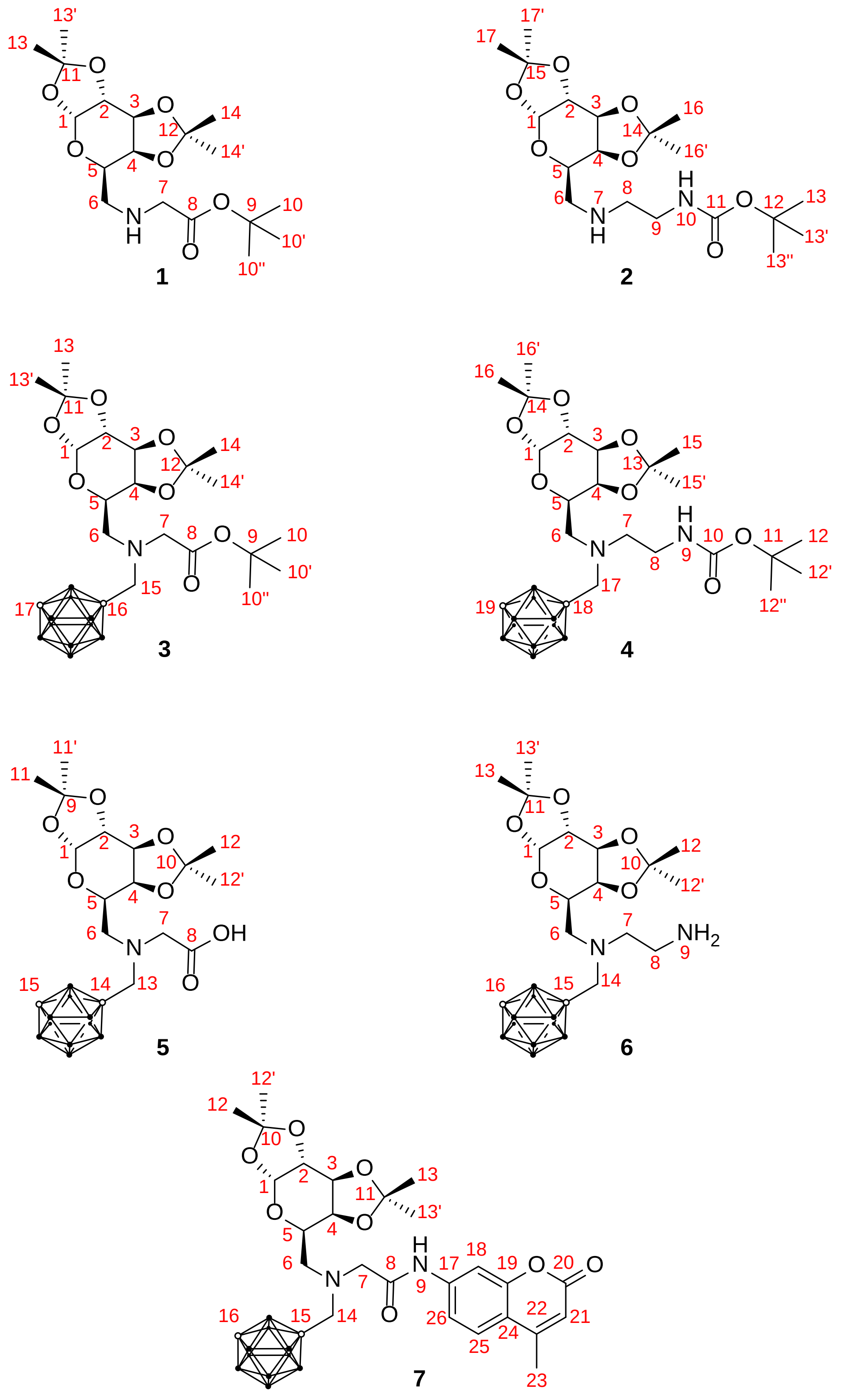

Figure S1: Numbering scheme of compounds **1** to **7**.

2. Depiction of the NMR spectra of compounds 5, 6, 7 and mass spectrum of 8 and 9

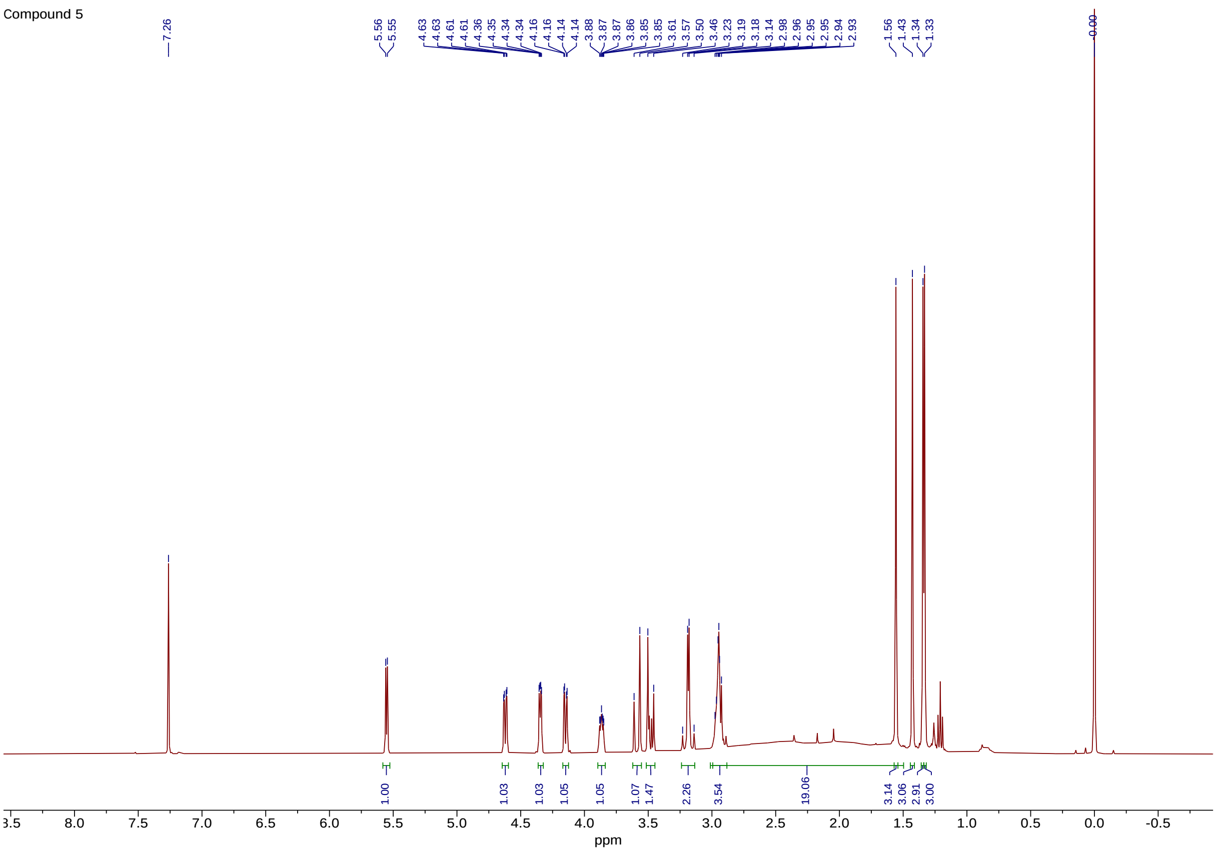

Figure S2: ^{1}H-NMR spectrum of compound **5**.

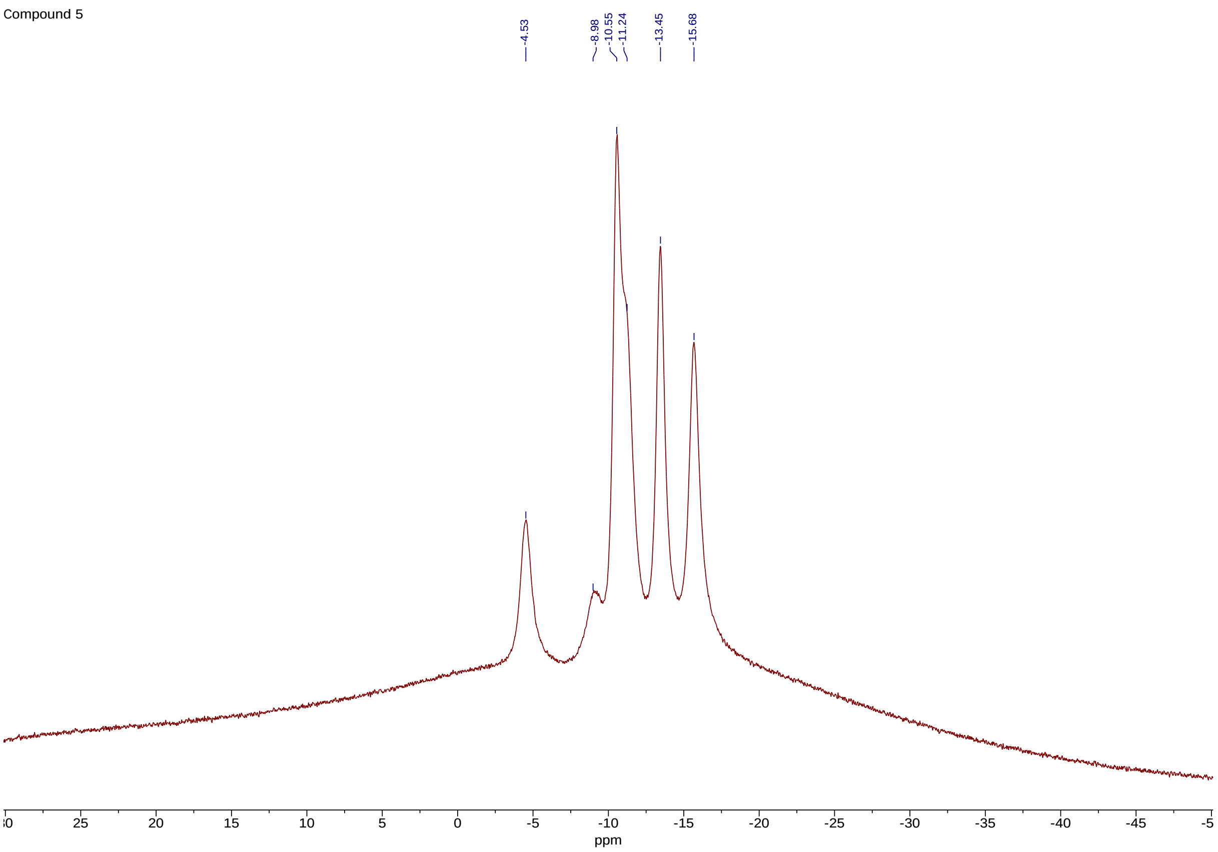

Figure S3: $^{11}B\{^{1}H\}$-NMR spectrum of compound **5**.

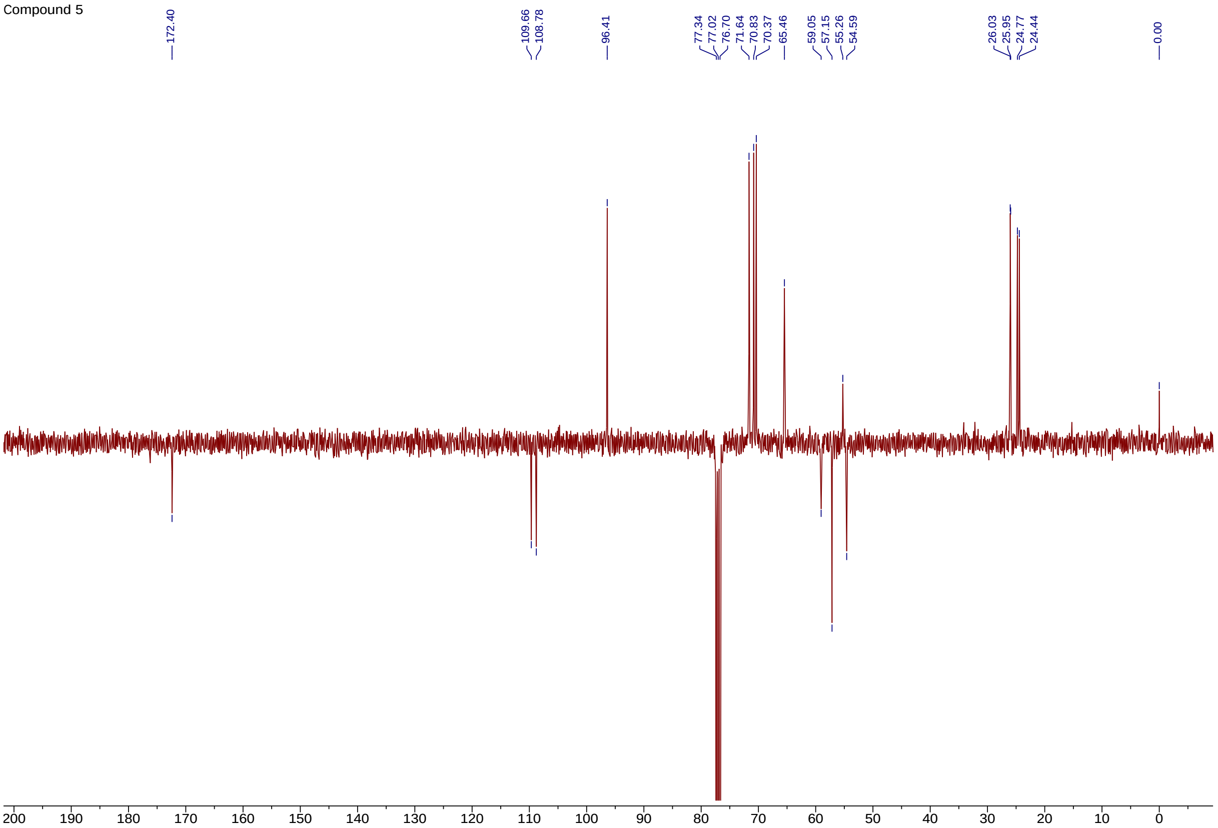

Figure S4: $^{13}C\{^{1}H\}$-NMR spectrum of compound **5**.

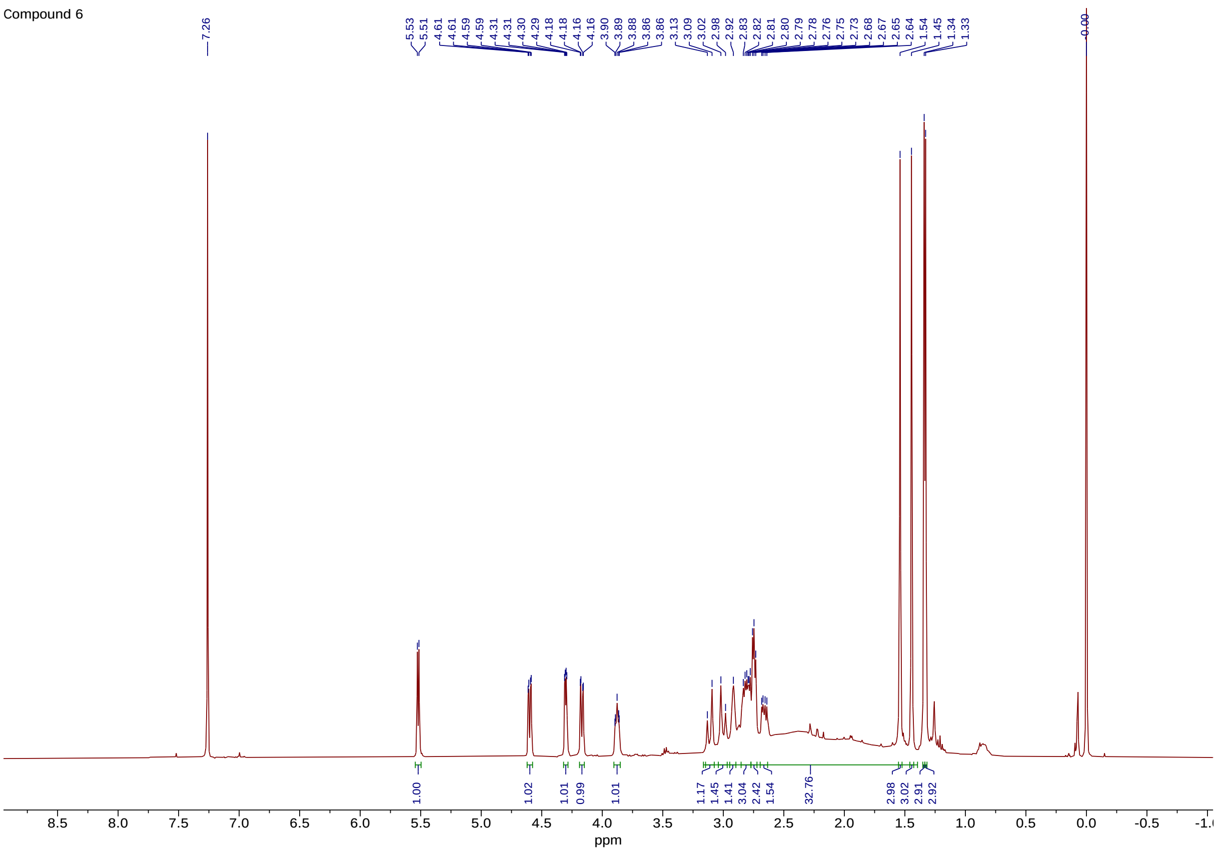

Figure S5: ^{1}H-NMR spectrum of compound **6**.

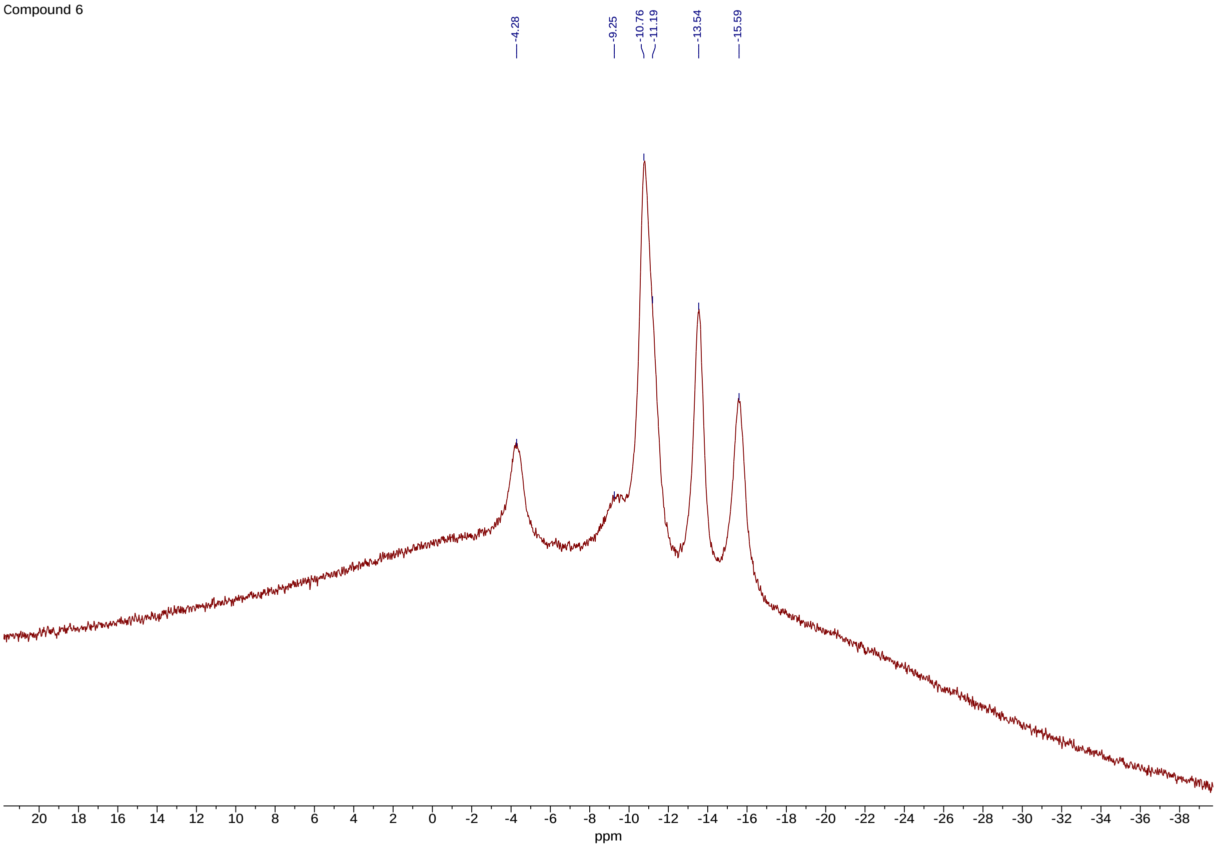

Figure S6: ^{11}B{^{1}H}-NMR spectrum of compound **6**.

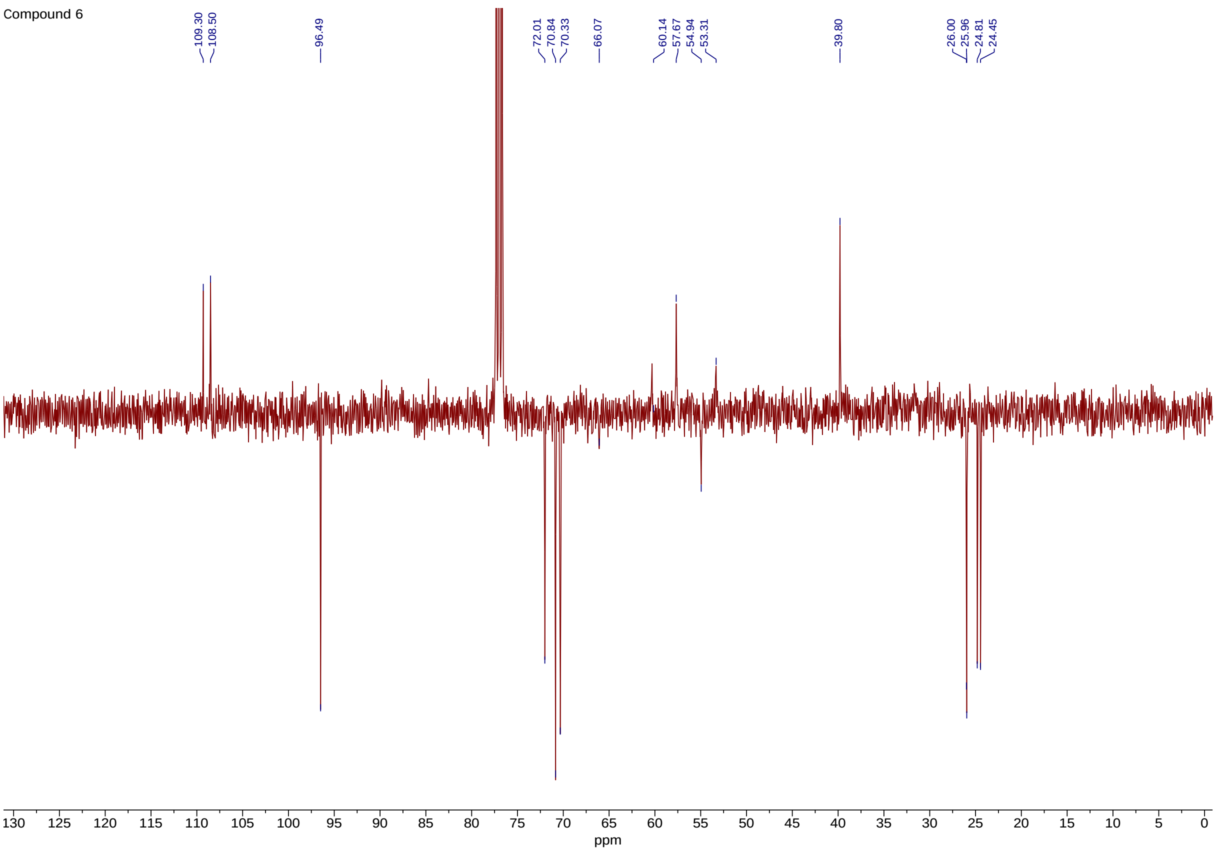

Figure S7: $^{13}C\{^{1}H\}$-NMR spectrum of compound **6**.

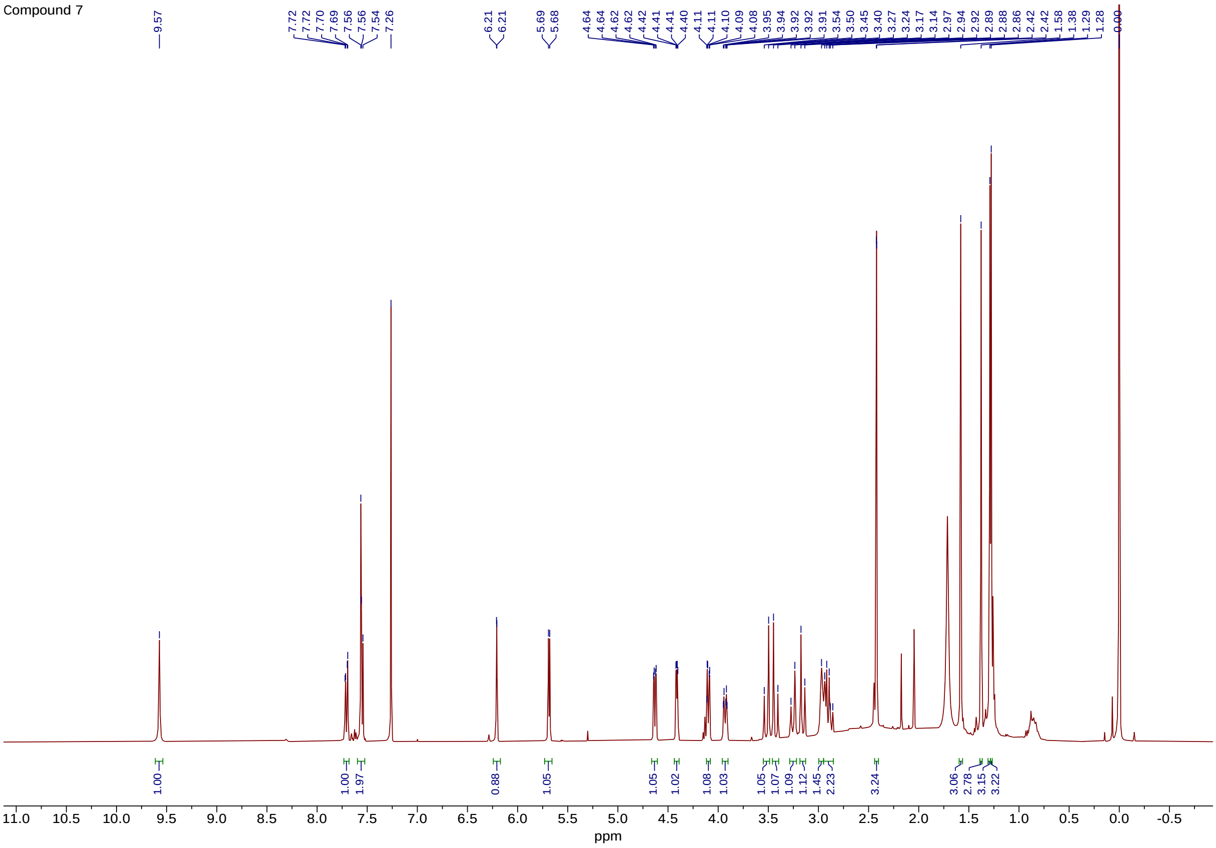

Figure S8: ^{1}H-NMR spectrum of compound **7**.

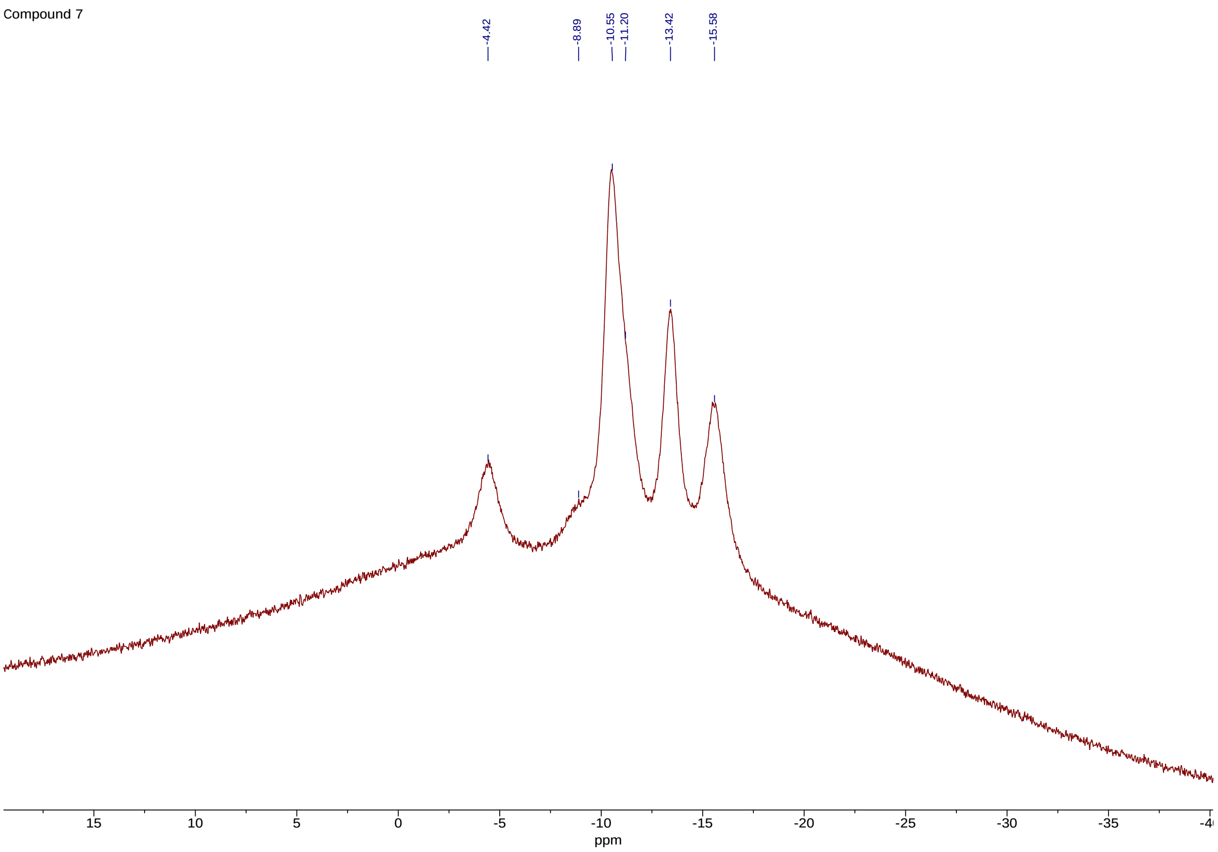

Figure S9: $^{11}B\{^{1}H\}$-NMR spectrum of compound **7**.

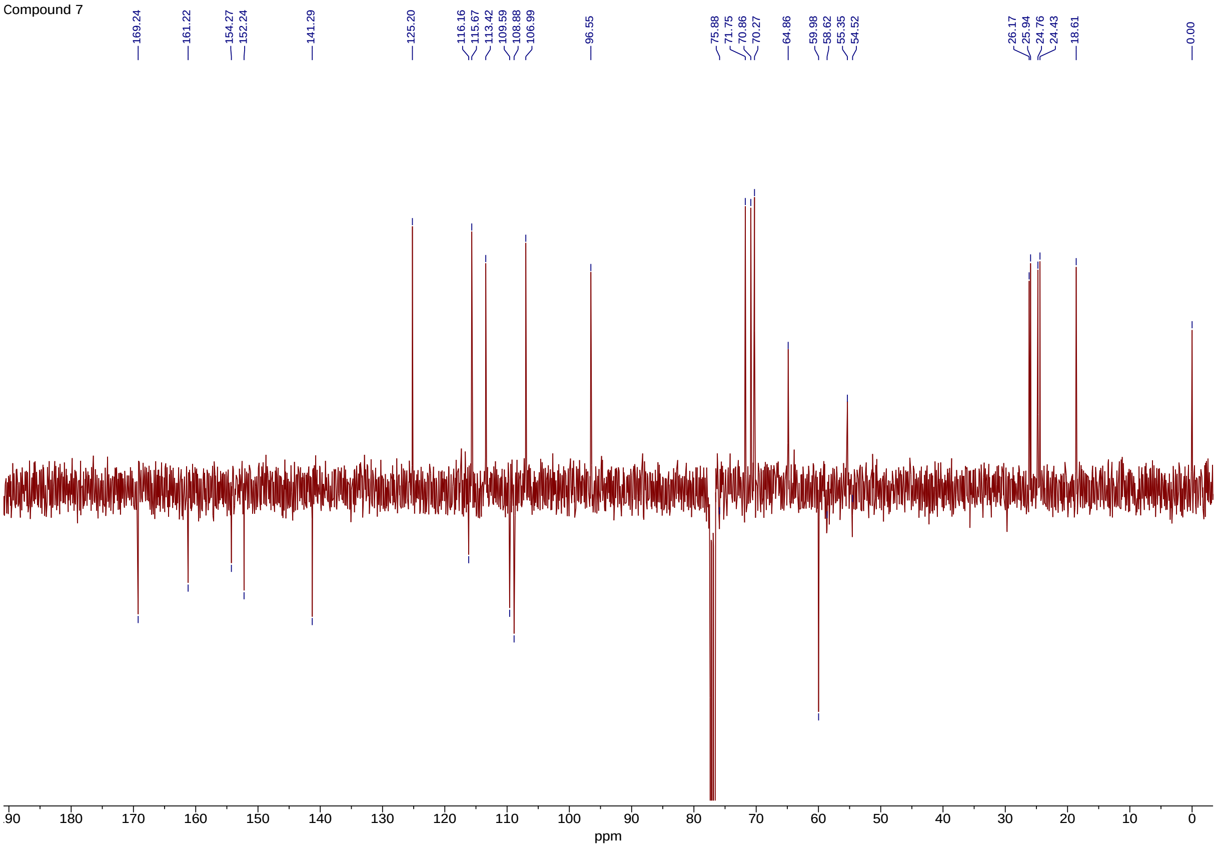

Figure S10: $^{13}C\{^{1}H\}$-NMR spectrum of compound **7**.

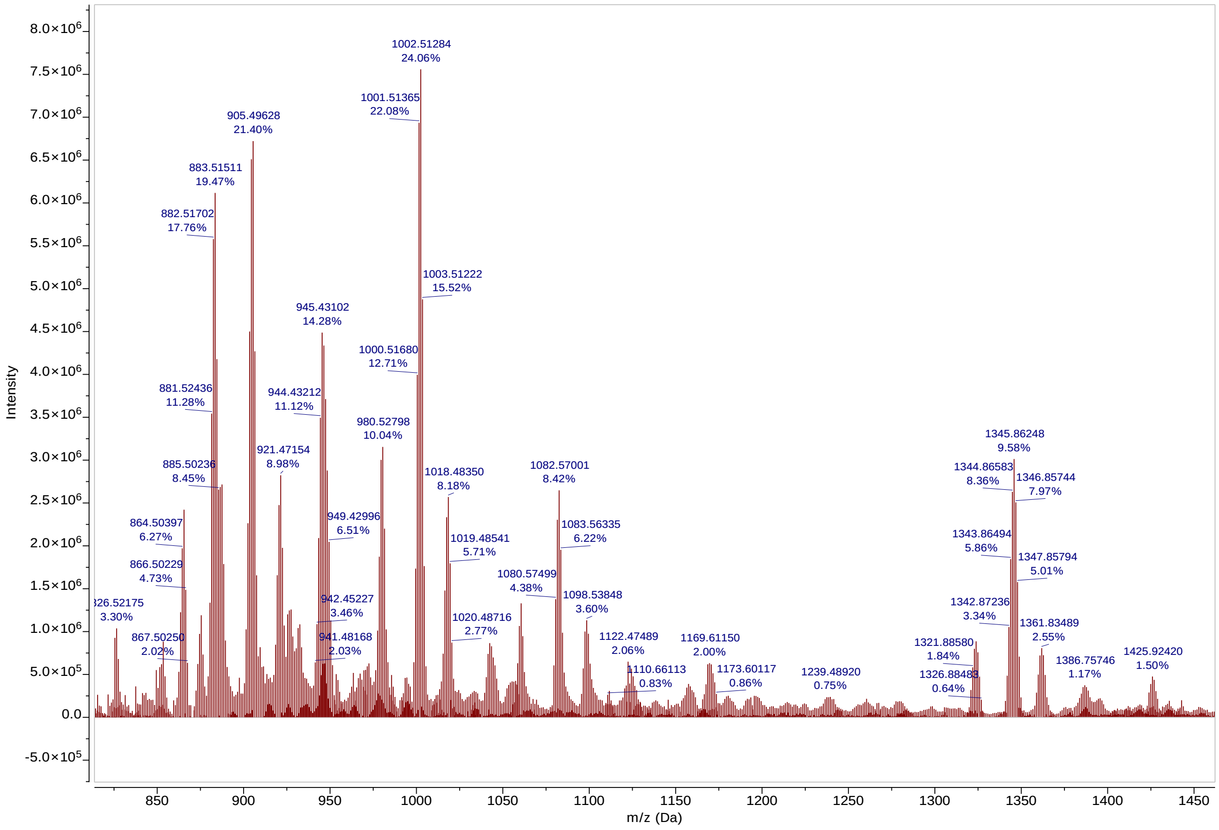

Figure S11: Mass spectrum of compound **8** and compound **9**.

3. Optimization of reaction conditions for the synthesis of 3

As described in the main text, the synthesis of compounds **3** and **4** (Figure S1) was carried out in toluene at 95 °C with potassium carbonate as base (Scheme 1, main text). As disubstitution of the protected ethylenediamine (to give ethylenediamine derivative **ESI-3'**) was not successful for carboranyltriflate **ESI-2** applying DIPEA in THF at room temperature (see below section 5, Table S3), the limiting factors were assumed to be the steric hindrance of the secondary amine after the first substitution with the carborane moiety and the reduced nucleophilicity of the resulting secondary amine **ESI-3** due to the electron-withdrawing effect of the carborane cluster. Therefore, the synthetic strategy was altered. First, a galactopyranosyl moiety was attached to the linking unit generating glycinate **1** in good yield (Scheme 1, main text), followed by reaction with 1-(trifluoromethanesulfonylmethyl)-1,7-dicarba-*closo*-dodecaborane(12). The optimized reaction conditions are given in Table S1.

Table S1: Optimization of reaction conditions to prepare the glycine-based tertiary amine **3**.

Entry	Solvent	Base	T [°C]	Time [h]	Yield [%]
**I**	MeCN	DIPEA	45 (48 h) then 55 (24 h)	72	19
**II**	MeCN	K_2CO_3	45	12	13
**III**	MeCN	K_2CO_3	45	24	16
**IV**	Toluene	K_2CO_3	95	43	54

A polar aprotic solvent and DIPEA at slightly increased temperatures (Entry I, Table S1) gave only low yield, as did a stronger base, namely potassium carbonate (Entry II and III). When the temperature was increased to 95 °C, a higher yield (54%) was observed (Entry IV). These reaction conditions were then also successfully applied for the synthesis of **4** (51% yield). Further optimization (solvent, temperature, base) was not attempted.

4. Optimization of the deprotection protocol for 4

According to the literature and our recent work, deprotection of glycinate esters and *tert*-butoxy carbamates can be carried out using excess trifluoroacetic acid (TFA) in dichloromethane with subsequent purification by column chromatography [1]. Using this procedure gave **3** and **4** is rather low yield. Therefore, in the case of carbamate **4**, the procedure was optimized (Table S2).

The reaction was carried out with 50.00 eq. TFA with dichloromethane as solvent (Entry I and III) and without any additional solvent (Entry II and IV). The mixture was stirred for two hours. To stop the reaction, all volatile components were removed under reduced pressure. Addition of dichloromethane to the resulting residue and repeated evaporation of all volatile components was performed four times (Entry I to IV). Since column chromatography is not suitable for this kind of polar compounds, two different work-up procedures were employed of which one proved to be the most effective.

<u>Method A:</u>

3 mL saturated sodium bicarbonate solution were added to the crude product. This mixture was sonicated for 15 min. The resulting floating precipitate was dissolved by adding 3 mL dichloromethane under observation of gas evolution. The mixture was stirred for 5 min. The aqueous layer was separated from the organic one. The organic layer was washed two times with distilled water (2 x 3 mL), dried over magnesium sulfate and the drying agent was filtered off. The solvent was removed under reduced pressure and product **6** (Scheme 1 main text or Figure S1) was isolated as a colorless foam.

<u>Method B:</u>

0.1 mL DIPEA (0.07 g, 0.55 mmol, 1.05 eq.) and 4 mL ethyl acetate were added to the crude product, the mixture was stirred for 5 min and then concentrated in vacuo to give a colorless precipitate. The precipitate was filtered off and washed two times with 1 mL ethyl acetate. The combined organic layers were concentrated and the product was isolated as a yellowish oil. Beside the product, DIPEA and ammonium salts were observed in the ^{1}H NMR spectra of this fraction, indicating insufficient purification using this method. Additional washing steps of the isolated product with distilled water did not result in a purer product.

Comparing both strategies, method A is the preferred method to obtain amine **6** in excellent yield.

Table S2: Optimization of the synthesis of **6**.

**Reagent**	**Carbamate 4**	**TFA**	**Solvent**	**t [h]**	**Purification method**	**η [%]**
**Entry I**	0.29 mmol<br>1.00 eq.	15.2 mmol<br>52.4 eq.	5 mL<br>CH_2Cl_2	**120**	Column*	n.d.
**Entry II**	0.54 mmol<br>1.00 eq.	26.0 mmol<br>48.2 eq.	/	**3**	Method A	quantitative
**Entry III**	0.52 mmol<br>1.00 eq.	26.0 mmol<br>50.00 eq.	4 mL<br>CH_2Cl_2	**2**	Method B	75
**Entry IV**	1.00 mmol<br>1.00 eq.	50.0 mmol<br>50.0 eq.	/	**2**	Method A	89

* column chromatography using isopropanol/*n*-hexane, 5:1 (v/v); n.d. – not determined

5. Side product *tert*-butyl-(2-{[bis(1,2:3,4-di-*O*-isopropylidene-6-deoxy-α-D-galactopyranos-6-yl)]-amino}ethyl)carbamate (2’)

During work up of compound **2**, the disubstituted compound **2’** was isolated as side product in 8% yield (with respect to the Boc-protected ethylenediamine as starting material) under the reaction conditions employed (MeCN, 40°C, DIPEA as base).

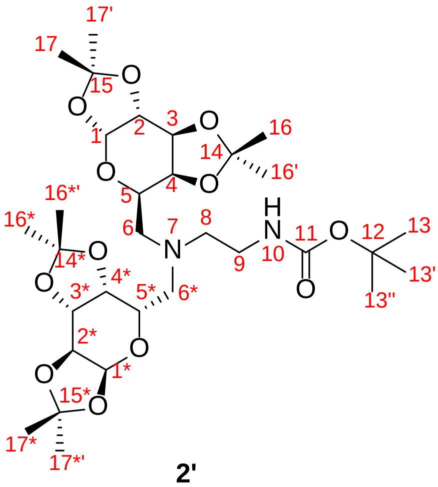

2'

Figure S12: Numbering scheme of compound **2’**.

^{1}**H-NMR** (400 MHz, $CDCl_3$): δ [ppm] = 1.31, 1.36, 1.43, 1.44, 1.52 (s, 33H, $^{13,13',13'',16,16*,16',16*',17,17*,\ 17'\ \text{and}\ 17*'}CH_3$), 2.35 (m, 1H), 2.50 (dd, 2H, $^{1}J_{HH}$ = 12.6 Hz, $^{3}J_{HH}$ = 5.3 Hz), 2.72 (m, 1H), 2.80 (dd, 2H, $^{1}J_{HH}$ = 12.6 Hz, $^{3}J_{HH}$ = 9.4 Hz), 3.10 (m, 1H), 3.31 (m, 1H), [Signals in the region from 2.35 to 3.31 ppm include ^{6}C, $^{6'}$C, ^{8}C and ^{9}C.], 3.96 (m, 2H, ^{5,5*}CH), 4.28 (dd, 2H, $^{3}J_{HH}$ = 5.1 Hz, $^{3}J_{HH}$ = 2.2 Hz, ^{2,2*}CH), 4.41 (dd, 2H, $^{3}J_{HH}$ = 8.1 Hz, $^{3}J_{HH}$ = 1.8 Hz, ^{4,4*}CH), 4.57 (dd, 2H, $^{3}J_{HH}$ = 8.1 Hz, $^{3}J_{HH}$ = 2.2 Hz, ^{3,3*}CH), 5.49 (d, 2H, $^{3}J_{HH}$ = 5.0 Hz, ^{1,1*}CH), 5.82 (s, br, 1H, ^{10}NH). **ESI-HRMS:** (*m/z*) calculated for $[C_{31}H_{53}N_2O_{12}]^+$ = 645.3554; observed 645.3609 $[M+H^+]^+$.

6. Extension of the synthetic protocol to *ortho*-carborane derivatives

The *ortho*-carborane as starting material was functionalized with a hydroxymethyl group (**ESI-1**), following the procedure of Nakamura and co-workers [2]. Subsequently, the hydroxy group was converted into a triflate group via the procedure of Kalinin and co-workers giving 1-(trifluoromethanesulfonylmethyl)-1,2-dicarba-*closo*-dodecaborane(12) (**ESI-2**) [3]. The next step was the combination of *tert*-butyl-*N*-(2-aminoethyl)-carbamate, the mono-Boc-protected ethylenediamine, with **ESI-2**. The primary amine exhibits the necessary nucleophilicity to undergo the desired reaction; in addition to the expected product *tert*-butyl-{2-[(1,2-dicarba-*closo*-dodecaboran-1-yl)methyl]aminoethyl}carbamate (**ESI-3**) a very small amount of a side product, namely *tert*-butyl-(2-{bis[(1,2-dicarba-*closo*-dodecaboran-1-yl)methyl]}aminoethyl)carbamate (**ESI-3'**), was observed. The last step was the deprotection of the *tert*-butoxycarbonyl protecting group under acidic conditions with trifluoroacetic acid (**ESI-4**) (Scheme S1).

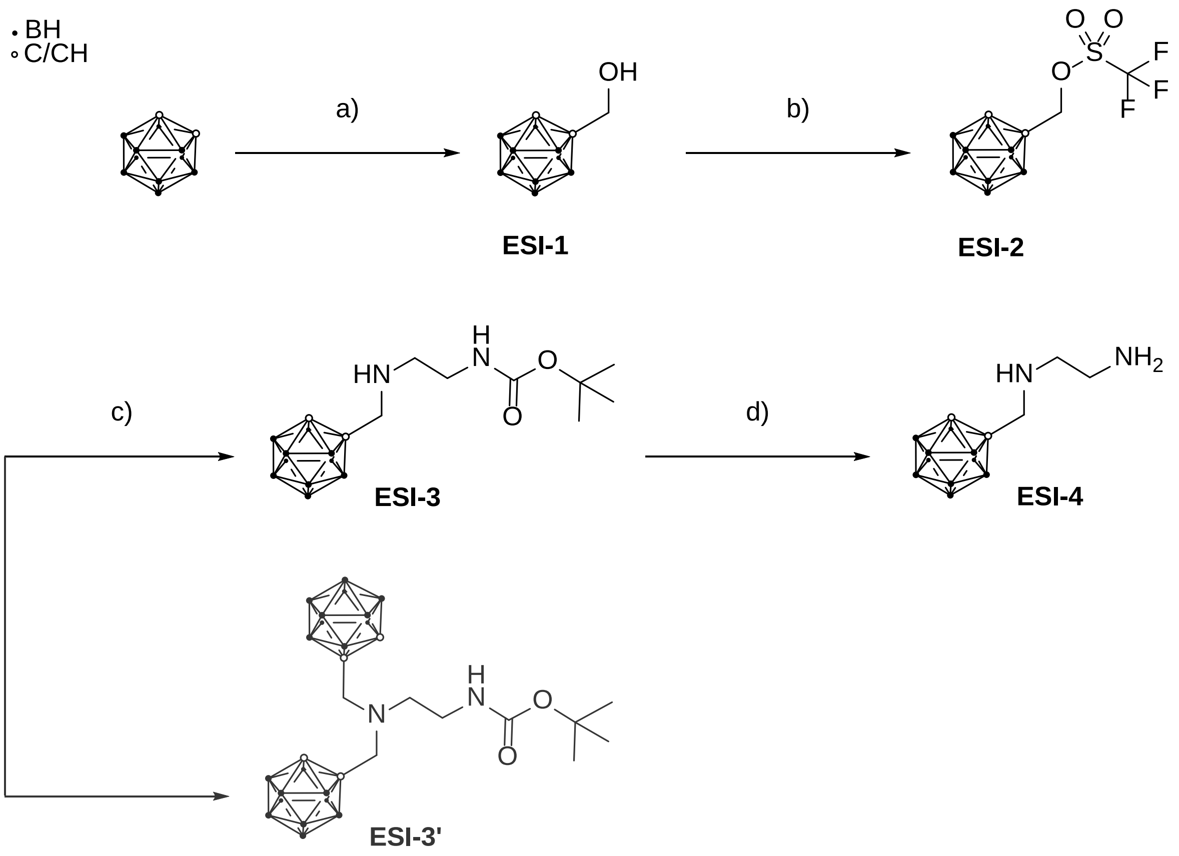

Scheme S1: Extension of the synthetic route developed for *meta*-carborane to 1,2-dicarba-*closo*-dodecaborane(12): a) 1.09 eq. formaldehyde (37% in H_2O), 2.95 eq. tetra-*n*-butylammonium fluoride trihydrate, THF, 30 min, rt, 42%; b) 1.00 eq. trifluoromethanesulfonic anhydride, 1.00 eq. pyridine, CH_2Cl_2, 3 h, rt, 89%; c) 1.00 eq. *tert*-butyl-*N*-(2-aminoethyl)-carbamate, 2.50 eq. *N*,*N*-diisoproylethylamine, THF, 42 h, rt, 93% up to quantitative yield; d) 19.1 eq. trifluoroacetic acid, CH_2Cl_2, 4 h, rt, 22%.

Table S3: Optimization of synthesis of **ESI-3**.

Entry	Eq. **ESI-2**	Eq. DIPEA	Reaction time [h]	η (**ESI-3**) [%]
I	1.5	1.5		94
II	2.0	2.5	42 to 44	95
III	3.0	3.0		quant.

The procedure was optimized using *N*,*N*-diisopropylethylamine (DIPEA) as base at room temperature in dry THF and varying stoichiometry (Table S3). Increasing the amount of triflate **ESI-2** should favor the formation of the disubstituted derivative **ESI-3’**. However, only a very small amount of the disubstituted product was obtained, and the main product in all three attempts was the secondary amine **ESI-3**. This observation shows the facile formation of the monosubstituted derivative **ESI-3** in excellent yield and good purity as well as the limited reactivity of both, the triflate **ESI-2** and the secondary amine **ESI-3**, to react further.

Pale-yellow prisms of **ESI-3’** suitable for single crystal X-ray structure determination were obtained from acetone. **ESI-3’** crystallized in the monoclinic space group $P2_1/n$ with one independent molecule in the asymmetric unit. The tertiary amine forms dimers with two hydrogen bonds between the NH and CO group of the *tert*-butoxycarbonyl protecting group (shown as dashed red lines) (Figure S13).

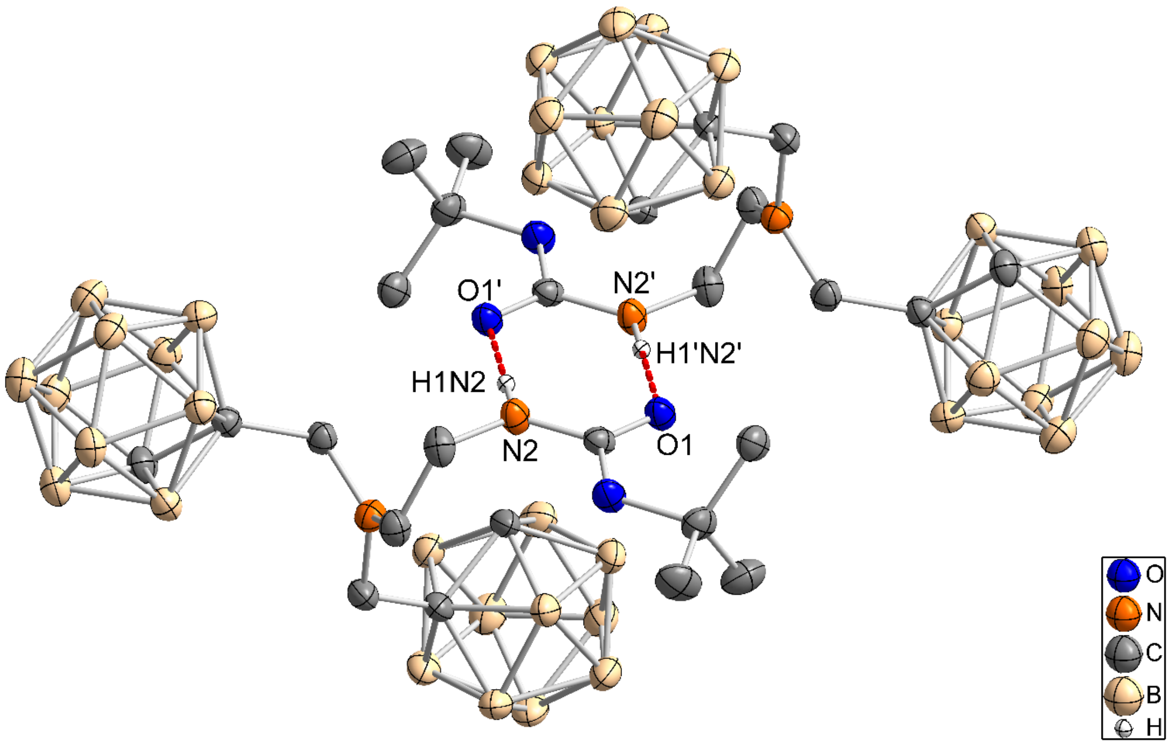

Figure S13: Molecular structure of the disubstituted ethylenediamine derivative **ESI-3’**. Hydrogen atoms which are not involved in hydrogen bonding are omitted for clarity. Hydrogen atoms participating in hydrogen bonding are shown with a fixed radius of 13.5 pm. Thermal ellipsoids are at the 50% probability level.

X-ray crystallography of ESI-3’

Table S4: Crystallographic data of compound **ESI-3’**.

Empirical formula	$C_{13}H_{40}B_{20}N_2O_2$	
Formula weight	472.67	
Temperature	130(2) K	
Wavelength	71.073 pm	
Crystal system	Monoclinic	
Space group	$P2_1/n$	
Unit cell dimensions	a = 1063.56(3) pm	α = 90°
	b = 985.84(2) pm	β = 98.765(2)°
	c = 2690.06(6) pm	γ = 90°
Volume	2.7876(1) nm^3	
Z	4	
Density (calculated)	1.126 Mg/m^3	
Absorption coefficient	0.059 mm^{-1}	
F(000)	992	
Crystal size	0.40 x 0.40 x 0.35 mm^3	
Theta range for data collection	1.972 to 30.166°	
Index ranges	$-13 \le h \le 14$<br>$-13 \le k \le 13$<br>$-37 \le l \le 32$	
Reflections collected	30980	
Independent reflections	7622 [R(int) = 0.0437]	
Completeness to θ = 28.285°	100.0 %	
Absorption correction	Semi-empirical from equivalents	
Max. and min. transmission	1.00000 and 0.97261	
Refinement method	Full-matrix least-squares on F^2	
Data / restraints / parameters	7622 / 0 / 494	
Goodness-of-fit on F^2	1.035	
Final R indices [I > 2σ(I)]	R_1 = 0.0519, wR_2 = 0.1264	
R indices (all data)	R_1 = 0.0827, wR_2 = 0.1422	
Largest diff. peak and hole	0.521 and –0.223 e·$Å^{-3}$	
CCDC deposition number	2 059 609	

Comments: Structure solution with SHELXT-2014 [4]. (dual-space method). Anisotropic refinement of all non-hydrogen atoms with SHELXL-2018 [5]. All H atoms were located on difference Fourier maps calculated at the final stage of the structure refinement. Carborane

carbon atoms could be localized from a bond length and displacement parameter analysis. A dimer is formed via intermolecular NH···O hydrogen donor acceptor bonds (Table S5).

Table S5: Intermolecular NH···O hydrogen donor acceptor bonds.

N–H···O	d(N–H) [pm]	d(H···O) [pm]	d(N···O) [pm]	<(NHO) [°]
N(2)–H(1N2) ···O(1)‡	84(2)	201(2)	284.8(2)	173(2)

‡: Symmetry transformations used to generate equivalent atoms: -x+1,-y+1,-z+1

6.1. Synthetic procedure and analytical Data of 1-(trifluoromethanesulfonylmethyl)-1,7-dicarba-*closo*-dodecaborane(12)

The procedure was conducted according to the reported, literature-known one for **ESI-2**, starting from 1-hydroxymethyl-1,2-dicarba-*closo*-dodecaborane [3].

Trifluoromethanesulfonic anhydride (2.10 mL, 12.7 mmol, 1.10 eq.), diluted in 5 mL dichloromethane, was added dropwise at 0 °C to a solution of 1-(hydroxymethyl)-1,7-dicarba-*closo*-dodecaborane(12) (2.00 g, 11.5 mmol, 1.00 eq.) and 2,4,6-collidine (1.53 mL, 11.5 mmol, 1.00 eq.) in 12 mL dichloromethane. The mixture was stirred at rt for 2 h and then diluted with dichloromethane. Subsequently, the organic layer was washed with H_2O (40 mL) and saturated NaCl solution (20 mL), the aqueous layer was extracted with dichloromethane (3 x 25 mL), the combined organic layers were dried over Na_2SO_4, the drying agent was filtered off and the solvent was removed under reduced pressure. The crude product was purified by column chromatography (*n*-hexane/ethyl acetate, 10:1 (v/v). Trifluoromethanesulfonylmethyl)-1,7-dicarba-*closo*-dodecaborane(12) was obtained as a colorless oil in 98% yield (3.13 g, 10.2 mmol, R_f = 0,56, *n*-hexane/ethyl acetate, 10:1 (v/v)). **^{1}H-NMR** (400 MHz, chloroform-d_1): δ [ppm] = 1.65 to 3.50 (br m, 10H, 10x BH), 3.04 (br s, 1H, CH), 4.53 (s, 2H, CH_2). **^{11}B{^{1}H}-NMR** (128 MHz, chloroform-d_1): δ [ppm] = –4.5 (s, 1B), –7.8 (s, 1B), –10.3 (s, 2B), –11.6 (s, 2B), –13.0 (s, 2B), –15.9 (s, 2B).

6.2. Synthetic procedures and analytical data of *tert*-butyl-{2-[(1,2-dicarba-*closo*-dodecaborane-1-yl)methyl]aminoethyl}carbamate (ESI-3) and *N*'-[(1,2-dicarba-closo-dodecaborane-1-yl)methyl]ethane-1,2-diamine (ESI-4)

***tert*-Butyl-{2-[(1,2-dicarba-*closo*-dodecaborane-1-yl)methyl]aminoethyl}carbamate (ESI-3):**

Method A: 548 mg (1.79 mmol, 2.00 eq.) 1-(trifluoromethanesulfonylmethyl)-1,2-dicarba-*closo*-dodecaborane(12) (**ESI-2**) were placed in a 100 mL round-bottom flask and 15 mL tetrahydrofuran were added. Subsequently, 0.38 mL (2.23 mmol, 2.50 eq.) diisopropylethylamine and then 0.14 mL (0.89 mmol, 1.00 eq.) *tert*-butyl-*N*-(2-aminoethyl)-carbamate were added dropwise via a syringe. The yellowish solution was stirred for 42 h at rt. The reaction was stopped by adding 15 mL saturated NH_4Cl solution. The resulting layers were separated, and the aqueous layer was extracted three times with 25 mL ethyl acetate each. The combined organic layers were washed once with 20 mL saturated NaCl solution, then dried over $MgSO_4$. The drying agent was filtered off and the solvent was removed under reduced pressure. The crude product was purified by column chromatography (*n*-hexane/ethyl acetate, 1:3 to 3:1, (v/v)). 270 mg (0.85 mmol, 95%) *tert*-butyl-{2-[(1,2-dicarba-*closo*-dodecaborane-1-yl)methyl]aminoethyl}carbamate (**ESI-3**) were isolated as a colorless oil. Additionally, 30 mg (0.06 mmol, 5%) *tert*-butyl-(2-{bis[(1,2-dicarba-*closo*-dodecaboran-1-yl)methyl]}aminoethyl)carbamate (**ESI-3'**) were isolated as a colorless solid. **ESI-3: ^{1}H-NMR** (400 MHz, $CDCl_3$): δ [ppm] = 1.44 (s, 9H, $(CH_3)_3$), 1.51 to 2.67 (m, 10H, 10x BH), 2.73 (t, 2H, CH_2, $^3J_{HH}$ = 5.8 Hz), 3.17 (q, 2H, CH_2, $^3J_{HH}$ = 5.8 Hz), 3.28 (s, 2H, CH_2), 4.06 (s, 1H, CH), 4.66 (s, 1H, NH). **^{13}C{^{1}H}-NMR** (100 MHz, $CDCl_3$): δ [ppm] = 28.4 (s, $(CH_3)_3$), 40.2 (s, NCH_2), 50.1 (s, NCH_2), 53.6 (s, NCH_2), 58.0 (s, CH), 75.1 (s, $C_{q,Cluster}$), 79.7 (s, C_q), 156.2 (s, C(O)O). **^{11}B{^{1}H}-NMR** (128 MHz, $CDCl_3$): δ [ppm] = –3.0 (1B), –5.5 (1B), –9.1 (2B), –11.5 (2B), –13.3 (4B). **ESI-HRMS** (*m/z*) calculated for $[C_{10}H_{29}B_{10}N_2O_2]^+$ = 317.3232; observed 317.3230 $[M+H]^+$; calculated for $[C_6H_{21}B_{10}N_2O_2]^+$ = 261,2606; observed 261,2600 $[M-[C_4H_9]^++2H^+]^+$. **IR** (KBr): ṽ = 3448 (w, vNH-sp^3), 3352 (m, vNH-sp^3), 2979 – 2928 (m, *v*CHsp^3), 2590 (s, *v*BH-sp^3), 1695 (s, *v*C=O), 1515 – 1018 (m), 859 (w), 781 (w), 724 (m, *v*BB-sp^3). **ESI-3': ^{1}H-NMR** (400 MHz, $CDCl_3$): δ [ppm] = 1.44 (s, 9H, $(CH_3)_3$), 1.50 to 2.87 (m, 20H, 20x BH), 2.94 (s, 2H, CH_2), 3.17 (m, 2H, CH_2), 3.57 (s, 4H, CH_2), 3.80 (s, 2H, CH), 4.65 (s, 1H, NH). **^{11}B{^{1}H}-NMR** (128 MHz, $CDCl_3$): δ [ppm] = –2.2 (1B), –4.8 (1B), –9.0 (2B), –11.8 (2B), –12.9 (4B). **ESI-HRMS:** (*m/z*) calculated for $[C_{13}H_{41}B_{20}N_2O_2^+]$ = 473.5174; observed 473.5177 $[M+H]^+$.

Method B: 855 mg (2.79 mmol, 3.00 eq.) 1-(trifluoromethanesulfonylmethyl)-1,2-dicarba-*closo*-dodecaborane(12) (**ESI-2**) were placed in a 100 mL round-bottom flask and 30 mL tetrahydrofuran were added. Subsequently, 0.48 mL (2.79 mmol, 3.00 eq.) diisopropylethylamine and then 0.15 mL (0.93 mmol, 1.00 eq.) *tert*-butyl-*N*-(2-aminoethyl)-carbamate were added dropwise via a syringe. The yellowish solution was stirred for 44 h at rt. The reaction was stopped by adding 30 mL saturated NH_4Cl solution. The resulting layers were separated and the aqueous layer was extracted three times with 25 mL ethyl acetate each. The combined organic layers were washed once with 30 mL saturated NaCl solution, dried over $MgSO_4$. The drying agent was filtered off and the solvent was removed under reduced pressure. The crude product was purified by column chromatography (*n*-hexane/ethyl acetate, 1:3 to 1:1, (v/v)). 341 mg (1.07 mmol, quant.) *tert*-butyl-{2-[(1,2-dicarba-*closo*-dodecaboran-1-yl)methyl]aminoethyl}carbamate (**ESI-3**) were isolated as a slightly yellow oil. Additionally, 145 mg (0.47 mmol, 17% of the starting material) 1-(trifluoromethanesulfonylmethyl)-1,2-dicarba-*closo*-dodecaborane(12) (**ESI-2**) were recovered as a colorless oil. Analytical data for **ESI-3** are the same as for method A.

***N'*-[(1,2-dicarba-closo-dodecaborane-1-yl)methyl]ethane-1,2-diamine (ESI-4):**

A 50 mL Schlenk flask was charged with 216 mg (0.68 mmol, 1.00 eq.) *tert*-butyl-{2-[(1,2-dicarba-*closo*-dodecaboran-1-yl)methyl]aminoethyl}carbamate (**ESI-3**) and dissolved in 4 mL dichloromethane. 1.00 mL (13.0 mmol, 19.1 eq.) trifluoroacetic acid was added dropwise to this mixture. The yellowish solution was stirred for 4 h at rt. The reaction was stopped by evaporation of all volatile components. The resulting crude product was dissolved in 2 mL dichloromethane and all volatile components were removed again under reduced pressure. This procedure was repeated twice. The resulting solid was treated with 3 mL distilled water (A) and the formed solid (B) was separated from the solution by filtration. The aqueous layer (A) was extracted three times with 5 mL dichloromethane each. The combined organic layers were dried over $MgSO_4$. The drying agent was filtered off and the solvent was removed under reduced pressure. The precipitate (B) was treated with 2 mL 1 M HCl, dissolved in 3 mL ethyl acetate and, subsequently, the acid was neutralized with saturated $NaHCO_3$ solution. This mixture was extracted twice with 4 mL dichloromethane. The combined organic layers were dried over $MgSO_4$. The drying agent was filtered off and the solvent was removed under reduced pressure. 10 mg of a mixture containing **ESI-4** were isolated from the original supernatant (A). From the original precipitate (B), 32 mg (0.15 mmol, 22%) *N'*-[(1,2-dicarba-*closo*-dodecaborane-1-yl)methyl]ethane-1,2-diamine (**ESI-4**) were obtained as a colorless solid. **^{1}H-NMR** (400 MHz, $CDCl_3$): δ [ppm] = 1.72 to 2.51 (m, 10H, 10x BH) 2.66 (t, 2H, CH_2, $^{3}J_{HH}$ = 5.6 Hz), 2.76 (t, 2H, CH_2, $^{3}J_{HH}$ = 5.6 Hz), 3.28 (s, 2H, CH_2), 4.11 (s, 1H, CH). **^{13}C{^{1}H}-NMR** (100 MHz, $CDCl_3$): δ [ppm] = 41.4 (s, NCH_2), 52.6 (s, NCH_2), 53.9 (s, $CH_{2,carborane}$), 57.9 (s, $CH_{carborane}$). **^{11}B{^{1}H}-NMR** (128 MHz, $CDCl_3$): δ [ppm] = –3.1 (1B), –5.5 (1B), –9.1 (2B), –11.4 (2B), –13.3 (4B). **ESI-HRMS:** (*m/z*) calculated for $[C_5H_{21}B_{10}N_2]^+$ = 217.2708; observed 217.2700.

7. References

1. Kellert, M.; Hoppenz, P.; Lönnecke, P.; Worm, D.J.; Riedl, B.; Koebberling, J.; Beck-Sickinger, A.G.; Hey-Hawkins, E. Tuning a modular system - synthesis and characterisation of a boron-rich s-triazine-based carboxylic acid and amine bearing a galactopyranosyl moiety. *Dalton Trans.* **2020**, *49*, 57–69, doi:10.1039/c9dt04031e.
2. Nakamura, H.; Aoyagi, K.; Yamamoto, Y. Tetrabutylammonium Fluoride Promoted Novel Reactions of o -Carborane: Inter- and Intramolecular Additions to Aldehydes and Ketones and Annulation via Enals and Enones. *J. Am. Chem. Soc.* **1998**, *120*, 1167–1171, doi:10.1021/ja973832e.
3. Kalinin, V.N.; Rys, E.G.; Tyutyunov, A.A.; Starikova, Z.A.; Korlyukov, A.A.; Ol'shevskaya, V.A.; Sung, D.D.; Ponomaryov, A.B.; Petrovskii, P.V.; Hey-Hawkins, E. The first carborane triflates: synthesis and reactivity of 1-trifluoromethanesulfonylmethyl- and 1,2-bis(trifluoromethanesulfonylmethyl)-o-carborane. *Dalton Trans.* **2005**, 903–908, doi:10.1039/b417199c.
4. Sheldrick, G.M. SHELXT: Integrated space group and crystal structure determination. *Acta Crystallogr.* **2015**, *A71*, 3–8.
5. Sheldrick, G.M. SHELXL. *Acta Crystallogr.* **2015**, *C71*, 3–8.
